# *Penicillium chrysogenum*, a Vintage Model with a Cutting-Edge Profile in Biotechnology

**DOI:** 10.3390/microorganisms10030573

**Published:** 2022-03-06

**Authors:** Francisco Fierro, Inmaculada Vaca, Nancy I. Castillo, Ramón Ovidio García-Rico, Renato Chávez

**Affiliations:** 1 Departamento de Biotecnología, Universidad Autónoma Metropolitana-Unidad Iztapalapa, Ciudad de México 09340, Mexico; 2 Departamento de Química, Facultad de Ciencias, Universidad de Chile, Santiago 7800003, Chile; inmavaca@uchile.cl; 3 Grupo de Investigación en Ciencias Biológicas y Químicas, Facultad de Ciencias, Universidad Antonio Nariño, Bogotá 110231, Colombia; nancastillo@uan.edu.co; 4 Grupo de Investigación GIMBIO, Departamento De Microbiología, Facultad de Ciencias Básicas, Universidad de Pamplona, Pamplona 543050, Colombia; rovigar@hotmail.com; 5 Departamento de Biología, Facultad de Química y Biología, Universidad de Santiago de Chile, Santiago 9170020, Chile; renato.chavez@usach.cl

**Keywords:** penicillin, *Penicillium chrysogenum*, *Penicillium rubens*, secondary metabolism, phylogeny of penicillin genes, transcriptional regulation, penicillin historical development, classical strain improvement, synthetic biology

## Abstract

The discovery of penicillin entailed a decisive breakthrough in medicine. No other medical advance has ever had the same impact in the clinical practise. The fungus *Penicillium chrysogenum* (reclassified as *P. rubens*) has been used for industrial production of penicillin ever since the forties of the past century; industrial biotechnology developed hand in hand with it, and currently *P. chrysogenum* is a thoroughly studied model for secondary metabolite production and regulation. In addition to its role as penicillin producer, recent synthetic biology advances have put *P. chrysogenum* on the path to become a cell factory for the production of metabolites with biotechnological interest. In this review, we tell the history of *P. chrysogenum*, from the discovery of penicillin and the first isolation of strains with high production capacity to the most recent research advances with the fungus. We will describe how classical strain improvement programs achieved the goal of increasing production and how the development of different molecular tools allowed further improvements. The discovery of the penicillin gene cluster, the origin of the penicillin genes, the regulation of penicillin production, and a compilation of other *P. chrysogenum* secondary metabolites will also be covered and updated in this work.

## 1. Introduction

*Penicillium chrysogenum* was not the first fungus ever to be used in an industrial process, this honor corresponds to *Aspergillus niger*, which, in 1919, was first put to produce a chemical for the benefit of people, citric acid [1]. However, *P. chrysogenum* does hold the honor of being the one that opened up the era of antibiotics, a milestone that would change medicine forever. Probably no other microorganism (and no other living being) has saved more human lives in history than this bluish green-coloured, velvety-surfaced, easy-to-disseminate filamentous fungus. We will use here his given name: *Penicillium chrysogenum*, although, a few years ago, it was reclassified (to the surprise of most involved) and renamed with the somewhat more prosaic name of *Penicillium rubens*.

*P. chrysogenum* still keeps a leading position in the biotechnology industry of pharmaceuticals and is one of the fungi on which more research is currently performed, having become a model for the study or secondary metabolism and its regulation. The availability of new molecular tools for its study and genetic manipulation and the arrival of the era of systems and synthetic biology has boosted the interest in this veteran performer, which, in spite of its long and very fruitful career, has apparently no intention to retire any time soon.

Here, we intend to tell a brief history of *P. chrysogenum*, focusing on aspects such as the historical development of improved strains, genomic studies of high-producing strains, the penicillin biosynthetic pathway and its regulation, the origin of the penicillin biosynthetic genes, other secondary metabolites produced by *P. chrysogenum*, metabolic engineering approaches, and synthetic biology developments. We will update these topics, linking the historical developments with the current state-of-the-art in penicillin and *P. chrysogenum* research, trying to give an overall comprehensive account of all of them.

## 2. A Star Is Born: From a Casual Discovery to the Limelight in Medicine, Industry, and Research

At present, the global penicillin production per year is around 50,000–60,000 MT [2], it accounted for the largest share (23.9%) in the antibiotics market as for 2020, and it is expected to dominate the market for the next years [3]. Penicillin is marketed in different forms, more usually as one of the naturally produced penicillins (penicillin G or V) to obtain by chemical or enzymatic conversion 6-aminopenicillanic acid (6-APA), which has become the world’s largest selling β-lactam bulk intermediate used as source of many over-the-counter marketed penicillin and other β-lactam semisynthetic derivatives [4]. Second to penicillin is another β-lactam antibiotic, cephalosporin, produced by *Acremonium chrysogenum* (formerly *Cephalosporium acremonium*) and discovered a few years later than penicillin [5,6]. For modern physicians, it is difficult to appreciate the enormous impact that the sudden availability of antibiotics meant for the practice of medicine in the 1940s and 1950s. One eyewitness at the time said “The crossing of the historic watershed could be felt at the time. One day we could not save lives, or hardly any lives; on the very next day we could do so across a wide spectrum of diseases” [7]. The absolute reductions in mortality afforded by antibiotics are virtually unparalleled in the annals of medical pharmacotherapy, and one of the major threats for the world’s health at present is actually the problem of antibiotic resistance [8].

It all started in August 1928, when Alexander Fleming returned from a vacation to his laboratory in St. Mary’s Hospital in London and observed something peculiar on one of the Petri dishes that had been set aside on the laboratory bench; in his own words: “It was noticed that around a large colony of a contaminating mould the staphylococcus colonies became transparent and were obviously undergoing lysis”, and he made a first attempt to classify the mould: “In its morphology this organism is a penicillium and in all its characters it most closely resembles *P. rubrum*” [9]. The name “penicillin” was initially assigned to the whole broth filtrate: “… for convenience and to avoid the repetition of the rather cumbersome phrase “Mould broth filtrate”, the name “penicillin” will be used” [9]. The potential of “penicillin” as a chemotherapeutic agent was quickly understood [10], and the first clinical use took place on 12 February 1941 on policeman Albert Alexander, aged 43, with a mouth and face suppuration that evolved into lung infection caused by *Staphylococcus aureus* and *Streptococcus pyogenes* [11,12]. The patient underwent a quick initial recovery upon several intravenous administrations of penicillin but died one month later due to lack of enough penicillin to complete the treatment. The next two cases were cured of postoperative wound infection with haemolytic *Streptococcus* and of carbuncle overinfected with *S. aureus*, respectively [11]. The authors concluded that: “Enough evidence, we consider, has now been assembled to show that penicillin is a new and effective type of chemotherapeutic agent, and possesses some properties unknown in any antibacterial substance hitherto described”, and added: “Evidence is produced of the low toxicity of the substance when applied directly to body tissues”. The main obstacle at that time was actually the difficulty to obtain enough purified penicillin, which was obtained from cultures in vessels with glazed interior where medium inoculated with the mould was introduced and recovered a few days later to extract the penicillin [11].

The mould isolated by Fleming would be reclassified as *P. notatum* Westling by Charles Thom, Principal Mycologist of the US Department of Agriculture (USDA) [13]. Different strains of *P. notatum* arrived at the Northern Regional Research Laboratory (NRRL) culture collection in Peoria, IL, from different sources. One of these strains, NRRL 1249, from the Squibb Institute for Medical Research, showed higher penicillin-producing capacity, and a derivative of this strain, 1249-B21, was chosen by the NRRL in 1943 for commercial production [14]. Production was initially performed in surface cultures, which would be soon substituted by the more efficient and controllable process of submerged aerated fermentation, where another strain, NRRL 832, unrelated to the Fleming isolate, outperformed NRRL 1249-B21 [13,14]. In 1943, the strain *P. chrysogenum* (Thom) NRRL 1951 was isolated from a cantaloupe “contributed by a housewife” in Peoria, which produced more penicillin than any of the *P. notatum* strains and was adopted as the strain for industrial production [15]. A strain improvement program was then initiated with this strain, which would raise penicillin productivity by more than 1000-fold [4,16,17].

The reason why *P. notatum* did not produce as high amounts of penicillin as *P. chrysogenum* was studied by Rodríguez-Sáiz et al. [18,19]. One of the precursors of penicillin G, phenylacetic acid (PAA), is catabolized through the homogentisate pathway, in which a cytochrome P450 enzyme encoded by the *pahA* gene participates. The authors found that, in *P. chrysogenum*, this gene contains a mutation (C^1357^→T, A394V) that drastically reduces the activity of this enzyme. When a *P. chrysogenum* high--producing strain was transformed with the *pahA* gene from *P. notatum*, the transformant acquired the capacity to catabolize PAA, and a 5-fold decrease in penicillin production was observed. The authors concluded that the decrease in PAA assimilation capacity was one of the most important genetic improvements that led *P. chrysogenum* to become the organism of choice for the industry of penicillin production. Some OMICs studies have been performed on the response of *P. chrysogenum* to the addition of PAA [20,21,22,23]. Recently, Pathak et al. [24] sequenced the genome of the original Fleming’s strain (*P. notatum* NRRL 824) and compared it with the *P. chrysogenum* reference strain (Wis. 54-1255) and an industrial strain (P2niaD18). The Fleming’s strain genome is only 0.106% divergent from the P2niaD18 genome. Penicillin-pathway genes show some degree of divergence in amino acid sequence between the Fleming strain and the *P. chrysogenum* strains, mainly the *pcbAB* and *penDE* genes, but the significance of these changes to the activity of the encoded enzymes is not known. No changes in the promoter sequences of the three biosynthetic penicillin genes were observed between *P. notatum* and *P. chrysogenum*; therefore, the regulation of the expression of the genes probably follows a similar pattern in both organisms.

Strain improvement programs aiming to increase penicillin yields starting from strain NRRL 1951 were carried out mainly by selection after random mutation with X-ray and ultraviolet radiation and chemicals such as chlormethine, nitrogen mustard, and nitrosoguanidine [14]. This process is known as classical strain improvement (CSI). In the first steps of CSI programs, carried out at the NRRL, Carnegie Institute, University of Minnesota and University of Wisconsin, strains such as NRRL 1951.B25, X-1612 and Wisconsin Q176 (Wis. Q176) were obtained, which gradually improved penicillin yields [14,15]. However, these strains produced large amounts of a yellow pigment that had to be removed from the broth in the process of penicillin purification; hence, efforts were taken to obtain strains devoid of this feature. An important step was the isolation in 1947 of strain Wis. BL3-D10, which did not produce the pigment and would be the parental strain in the development of various CSI programs in different laboratories [14]. As a result of these programs, strain Wis. 54-1255 was obtained at the University of Wisconsin. This strain has been widely distributed and has become the standard for research on penicillin biosynthesis and other biochemical, genetic, and cellular processes of *P. chrysogenum*. Its genome was the first to be sequenced and has been used as reference for molecular biology and OMICs studies of the fungus [20]. Some events leading to the increased capacity of penicillin production in strain Wis. 54-1255 were described by van den Berg [25].

Once penicillin started to be successfully used as antibacterial treatment at the beginning of the 1940s, several companies became interested in its production and commercialization. Backus and Stauffer [14] reported that: “Released late in 1945, strain Wis. Q176 was widely sought by commercial producers of penicillin, perhaps as much for use as a “breeding stock” in their own strain development programs as for use in their production plants (…). Over 150 soil preparations of strain Q176 have been distributed by the writers in response to requests from industrial laboratories, university laboratories (…) etc. in nineteen countries”. The exact origin of the strains that would be used by companies such as Panlabs Inc., Smith Kline Beecham Pharmaceuticals, or Antibióticos S.A. to develop their own strains for industrial production is not known. The literature generally says that they come from strains early derived from NRRL 1951. The information provided by Backus and Stauffer [14] suggests that at least some of these strains may derive from Wis. Q176. In the case of strains of DSM Biotechnology (formerly Gist-brocades, Delft, The Netherlands), such as DS17690, they are described as derived from Wis. 54-1255 [26].

Random mutagenesis causes different types of mutations in the genome, which eventually may result in the improvement of the performance of the treated strain. The kind of genetic changes and mutations underwent by high penicillin-producing strains would be gradually unveiled as genetic analysis techniques became available, techniques that also allowed the identification of the biosynthetic genes and subsequently other auxiliary and regulatory genes involved in penicillin production. The advent of the OMICs era provided the ultimate tools for the in-depth analysis of the genetic and functional changes caused by mutations during the CSI programs. Some of the strains developed by companies for industrial production have recently had their genomes sequenced [26,27,28]. In addition, transcriptomic, proteomic, and metabolomic studies have been performed trying to elucidate the metabolic changes underwent by industrial strains that led to the high productivity they possess [20,21,26,29,30,31,32,33,34,35]. Some recent reviews summarize and make a comprehensive account of the main results obtained in these studies [25,36,37,38,39,40].

## 3. À la Recherche de la Mutation Perdu

The genetic and genomic studies performed on high-producing strains obtained in CSI programs reveal the presence of different kinds of mutations in their genomes that have contributed to their improved penicillin yields. These mutations can be grouped into three types of genetic changes: chromosomal rearrangement, amplification of the penicillin gene cluster, and point mutations.

### 3.1. Chromosomal Rearrangement

One of the genetic changes caused by mutagenesis is chromosome translocations, which were first detected using pulsed field gel electrophoresis (PFGE) to separate entire chromosomes of strains with different penicillin production capacity (NRRL 1951, Wis. 54-1255, AS-P-78 from Antibióticos S.A. and P2 from Panlabs Inc.) [41]. The occurrence of chromosomal rearrangement is common during mutational treatments applied to fungal strains for production improvement, as shown also for an *Aspergillus nidulans* strain (ATCC 28901) obtained after UV irradiation and with improved penicillin production [42] and for different *A. chrysogenum* strains with higher production of the β-lactam antibiotic cephalosporin [43,44,45,46]; in all these examples, the chromosomal rearrangements were detected by PFGE. For a discussion on mechanisms of chromosomal rearrangement in fungi, see Fierro and Martín [47]. A more precise technique to elaborate karyotypes and estimate chromosome sizes (physical mapping by restriction fingerprinting aided by capillary electrophoresis) was applied to *P. chrysogenum* Wis. 54-1255 by Xu et al. [48], obtaining results very similar to those obtained by PFGE for this strain.

Analysis of the genomes of industrial strains obtained by CSI has always revealed chromosomal rearrangement. Specht et al. [27] obtained the complete genome sequence of strain P2niaD18, derived from strain P2 after UV treatment [49]. Two large chromosomal translocations were found between strains Wis. 54-1255 and P2niaD18, one of them involving chromosomes II and III and the other chromosomes II and IV, the first one causing nitrate reductase deficiency due to splitting of the *niaD* gene. Specht et al. [27] also reassembled the genomic sequence of strain Wis. 54-1255 previously obtained by van den Berg et al. [20], establishing accurate sizes for the chromosomes, which are in good accordance with those estimated by PFGE for this strain [41]. Wang et al. [28] obtained the genomic sequence of strain NCPC10086, provided by the North China Pharmaceutical Group Corporation, and also found two large translocations. One of them was a 266 kb fragment translocated from its original subtelomeric position in strain Wis. 54-1255 to a near centromeric position in NCPC10086; it contains 107 genes, whose function is not known in most cases. One of the genes in this fragment is *nre*, encoding the nitrogen metabolite repression regulator. The other translocation corresponds to a 1202 kb fragment, which contains 494 genes, some of them associated with energy metabolism and the peroxisome pathway.

Chromosome rearrangement has also been found when comparing wild strains between them and with strains obtained in CSI programs. In strain P2niaD18, Böhm et al. [50] detected at least one chromosomal rearrangement when compared to the wild strain Pc3. Sequences from contigs 24 and 35 of the Pc3 genome sequence had been translocated to opposite ends of chromosome I in P2niaD18. Wong et al. [51] analyzed the wild strain PC0184C and Wis. 54-1255 in search for rearrangements and found 10 large insertion/deletion events (>1 kb) and four inversions in gene-rich regions. Interestingly, the position of the rearrangements was biased towards regions including or proximal to penicillin-related genes (*p* = 0.038), and thus they could be involved in the higher production of strain Wis. 54-1255. Perhaps the most interesting finding in the work of Wong et al. [51] is that most of the genomic rearrangements present in strain Wis. 54-1255 and associated to penicillin-related genes are segregating in several wild strains analyzed. The starting hypothesis was that if chromosomal rearrangements occurred during CSI, such structural changes should be specific to the Wis. 54-1255 genome. However, for most of the rearrangements that distinguished strain PC0184C from Wis. 54-1255, the latter resembled its wild progenitor, strain NRRL 1951. This result strongly suggests that most of the differences in chromosome structure between the PC0184C and Wis. 54-1255 strains that may have affected penicillin production would be ancient and segregating in wild *P. chrysogenum* populations. To confirm this hypothesis, the authors analyzed the 14 rearrangements in four additional wild isolates: the original Fleming strain (NRRL 824), NRRL 832 (one of the first isolates in the initial NRRL program, see above), and two recently isolated wild strains (Henk PC08-3A and NRRL A3704). The results showed that almost all the rearrangements were polymorphic across this set of strains, which indicates that chromosomal rearrangements are common in wild populations and, apparently, there is a preference for regions proximal to genes involved in penicillin biosynthesis. Therefore, many of the genome structural features that predisposed for high penicillin production were already in place in the cantaloupe-isolated strain NRRL 1951. Natural selection had already done its part in selecting for high penicillin producers, they were out there, and the anonymous “housewife” who contributed the famous cantaloupe, as recounted by Raper and Alexander [15], seems to have had a very good eye to pick up precisely the one harbouring the now celebrated NRRL 1951 strain.

### 3.2. Amplification of the Penicillin Gene Cluster

Amplification of the penicillin biosynthetic genes in high-producing strains was first discovered by Barredo et al. [52] and Smith et al. [53]. This finding made it clear that one of the phenomena leading to increased penicillin production in the strains obtained by CSI was gene dosage. Subsequently, the amplified region containing the penicillin genes was characterized [54]. Two different amplified regions were found, a long one of 105.2 kb in strains P2 and AS-P-78 and a shorter region of 56.9 kb in strain AS-P-E1. In both cases, the copies of the amplified region were arranged in head-to-tail tandem repeats. Strain P2 belongs to the Panlabs strain improvement program [17], and the other two were obtained at Antibióticos S.A. (León, Spain). Since then, a number of industrial high-penicillin-producing strains have been analyzed, some of them by genome sequencing, and in all cases, tandem amplification of the same 56.9 kb region has been demonstrated, but for strain P2niaD18, derived from P2, where the amplified fragment is 105.2 kb long, as expected. In some cases, amplification of the penicillin cluster in industrial strains has been proven but the extent of the amplified region was not determined. Smidák et al. [55] reported that the entire penicillin cluster is present in four copies in strain NMU2/40 and in six copies in strain B14, both strains provided by Biotika a.s., Slovenska Ľupča (Slovakia).

The amplification of the penicillin gene cluster is logically related to the increased production of penicillin in industrial strains. Several studies have been conducted to determine how these two variables are related. In general, amplification and penicillin production are directly correlated up to a certain number of repeats [56,57,58], beyond which the correlation is lost [57,58], other factors probably entering the scene then such as regulatory processes or availability of precursors. In contrast, correlation between copy number of the penicillin cluster and penicillin production can be absent even at low copy numbers in some high-producing strains, as shown by Ziemons et al. [59], who reported that the loss of one of the two copies present in strain P2niaD18 had no consequences in the level of produced penicillin. This result suggests that, in this strain, other factors override the gene dosage effect regarding penicillin production.

### 3.3. Point Mutations Targeting Secondary Metabolism Genes, Regulators, and Other Genes

In addition to the major genetic changes described in the previous sections, point or small mutations occurring during the CSI programs can also have an important effect on penicillin production. The development of comparative genomics has allowed to detect with precision those mutations and, sometimes, infer how they affect production. In general, it has been observed that the biosynthetic genes have not been targeted by mutations; instead, regulatory mechanisms, supply of precursors and competing pathways of other secondary metabolites have been shown to be targets of mutations.

Salo et al. [26] compared the genomic sequence of NRRL 1951, Wis. 54-1255, and the industrial strain DS17690, and in addition, they performed metabolome and transcriptome profiling. The most interesting findings of this study were the loss of production of a yellow pigment present in wild strains (commented above), the mutations in genes of secondary metabolism, and the mutations affecting components of the Velvet complex. Mutations in genes encoding polyketide synthases (PKS) were frequent in the strains submitted to CSI. Two of these mutations, in the PKS12- and PKS13-encoding genes, which form a cluster with other five genes, were responsible for the loss of the yellow pigment, identified as a mixture of sorbecillinoid molecules. In total, eight PKS genes and one NRPS (non-ribosomal peptide synthase) gene were mutated in strain DS17690, from a total of 20 PKS and 11 NRPS genes present in the genome, which indicates that secondary metabolite pathways competing with the penicillin pathway have been targeted during CSI programs. Regarding regulatory genes, Salo et al. [26] found that the Velvet complex has been targeted by mutations. The *laeA* gene accumulated two mutations in strain DS17690, while the *velA* gene was mutated once, which resulted in a truncated VelA protein. This would affect assembly of the Velvet complex, which consequently would be functionally impaired in this strain. The Velvet complex has an important role in the regulation of development and secondary metabolism [60], and thus these mutations may have been relevant for the increased penicillin production capacity of strain DS17690.

### 3.4. Concluding Remarks on the Genetic Analysis of Strains Obtained in CSI Programs

Amplification of the penicillin gene cluster, chromosomal rearrangement involving regions with presence of penicillin-related genes, and point mutations in genes involved in global regulation (Velvet complex) and secondary metabolite pathways are the main genetic changes found in high-producing strains detected by genetic and genomic analysis. Other OMICs methodologies, such as proteomics and transcriptomics, allow more functional approaches to elucidate how the metabolism and other cellular processes have been affected during CSI programs to increase penicillin productivity (reviewed in [25,36,37,38,39,40]). As a quick summary, we can mention that, in high-producing strains, there is an upregulation of proteins involved in synthesis of NADPH and cysteine as well as in ATP-producing carbon catabolism, the three compounds having proven essential for high levels of penicillin production [61,62]. For their part, proteins involved in the synthesis of some secondary metabolites other than penicillin and virulence-related proteins appear downregulated. There is also an increase in the abundance of proteins related to stress response [31], highlighting the importance of stress signals for triggering secondary metabolism/penicillin production, and proteins related to peroxisomes, which are more abundant in high-producing strains [30]. As mentioned above, the Velvet complex has been targeted during CSI programs and, as a consequence, genes regulated by this complex show important changes in expression between low- and high-penicillin-producing strains [35].

The arrival of the genetic engineering era with the subsequent development of an array of tools for genetic manipulation entailed a leap forward in the development of strain improvement programs, incorporating approaches of metabolic engineering, a topic that will be treated in a later section. With all the knowledge gathered so far from genomics and other OMICs studies, it is now possible to design more accurate and directed strategies to improve β-lactam antibiotics production, knowledge that may well be extensible to the production of many other fungal natural products.

## 4. *Penicillium chrysogenum* Reveals a Secret and Changes Identity

By 2008 *P. chrysogenum* was one of the most studied fungi; exhaustive work had been performed with it in both CSI programs and genetic engineering. However, it still kept a big secret, which began to be unveiled by Hoff et al. [49]. In ascomycetes, mating usually occurs between partners carrying dissimilar DNA sequences at the mating-type (MAT) locus: MAT1-1 and MAT1-2. An alpha-box domain-containing protein is encoded by MAT1-1, whereas a protein with a high-mobility group (HMG) domain is encoded by MAT1-2 [63,64,65]. Heterothallic ascomycetes (obligate outcrossing) carry one of the two idiomorphs, MAT1-1 or MAT1-2, which determine the “sexual compatibility” of the corresponding strains [66].

### 4.1. P. chrysogenum Contains Functional Mating Type Genes

*P. chrysogenum* was thought to lack a sexual cycle, and because of this, it was traditionally placed in the vague taxonomic category of “imperfect” fungi or Deuteromycota. Upon genome sequencing of two close relatives of *P. chrysogenum*, the opportunistic pathogens *Aspergillus fumigatus* and *Penicillium (Talaromyces) marneffei*, it was revealed that they contained sexual reproduction-associated genes such as mating-type genes and genes for pheromone production and detection [67,68]. Hoff et al. [49] used primers belonging to conserved flanking regions of the *A. fumigatus* MAT locus to amplify this region from 12 *Penicillium* strains, among them the original Fleming isolate (NRRL 824) and the NRRL 1951 strain. Two different amplicons were obtained, with sizes of 3.6 and 3.7 kb. Six strains, including NRRL 1951, generated an amplicon of 3.7 kb, while the other six, including the Fleming strain, generated a smaller 3.6-kb amplicon. Sequencing of the larger amplicon revealed the presence of a putative 1077-bp *mat1-1-1* gene encoding a predicted protein of 342 amino acid residues containing a conserved alpha-box domain, while the smaller amplicon contained a 1136-bp ORF encoding a 303-amino acid protein with an HMG-DNA-binding domain. Highly conserved sequences (95.8% identical nucleotides) were also found flanking the MAT1-1 idiomorphs. No inactivating mutations were present in the genes encoding the alpha-box and HMG-domain proteins, and RT-PCR revealed that both genes were expressed. Homologues of genes encoding pheromones (*Pcppg1*) and pheromone receptor genes (*Pcpre1* and *Pcpre2*) that participate in mating of sexually reproducing ascomycetes were also found in the *Penicillium* strains of both mating-types. These genes were also shown to be transcriptionally active. *Pcpre1* encodes a protein with high identity with the *Saccharomyces cerevisiae* a-factor receptor Ste3p, whereas *Pcpre2* encodes a protein with high identity to the α-factor receptor Ste2p. From these results, Hoff et al. [49] concluded that that *P. chrysogenum* NRRL 1951 and *P. notatum* (NRRL 824) are opposite mating types (MAT1-1 and MAT1-2, respectively), probably within the same heterothallic species. Other β-lactam producing fungus, the cephalosporin producer *A. chrysogenum*, was also found to contain a functional mating locus in its genome [69]. A number of presumed asexual fungi were found to actually contain functional mating loci, and in some cases, mating or sexual reproduction could be confirmed [70]. It seemed as if, decades after that of humans, fungi were experiencing their own “sexual revolution” at the time, in the words of Dyer and O’Gorman [71].

### 4.2. P. chrysogenum Is Reclassified as P. rubens

The presence of apparently functional genes for sexual crossing was not the only surprise awaiting during those years. Houbraken et al. [72] revealed that both *P. chrysogenum* NRRL 1951 (and all its derivatives) and *P. notatum* (NRRL 824) should be reclassified as *Penicillium rubens*. *P. chrysogenum* is a commonly occurring mould in indoor environments such as dust, indoor air, and damp building materials as well as in decaying food. *P. chrysogenum* was first described by Thom [73]; the taxonomy of this species was studied extensively by Raper and Thom [74], and they accepted four species in the “*Penicillium chrysogenum* series”, one of them named *P. notatum*. Later, more species were included in the Penicillium series Chrysogena, although they are phenotypically different, but penicillin production is common to all of them (see Houbraken et al. [72] and references therein). Scott et al. [75] studied the taxonomy of *P. chrysogenum* using multilocus sequence typing (MLST) data and showed that it could be subdivided into four clades. Houbraken et al. [72] then performed a study to find out whether the two most common clades in *P. chrysogenum sensu lato* [75] represent one or two species and analyzed the most important strains in the history of penicillin production using extrolite and molecular data. The molecular data revealed two highly supported clades in *P. chrysogenum sensu lato*. One clade was centered around several strains including the ex-type strain of *P. notatum* (CBS 355.48), while the other was centered around the ex-type strain of *P. rubens* (NRRL 792). Fleming’s original isolate (CBS 205.57 = NRRL 824), strains NRRL 1951, Wis. 54-1255, and the first strain producing satisfactory penicillin yields in submerged culture (CBS 197.46 = NRRL 832), were all accommodated in the latter clade. It was concluded that the two clades represent two different species: *P. chrysogenum* and *P. rubens*. Members of the two species are phenotypically very similar and difficult to distinguish, but extrolite analysis revealed a clear difference: *P. chrysogenum sensu stricto* produces secalonic acid D and F and/or a metabolite related to lumpidin, whereas *P. rubens* does not produce these metabolites.

Although it is common belief that A. Fleming misidentified his fungus as *P. rubrum* and was later rectified by Charles Thom who would have correctly identified it as a *P. notatum*, the story is more convoluted. It was actually the experienced mycologist Charles J. La Touche, working in a room on the floor below that occupied by Fleming, who identified the strain as *P. rubrum*, and he might have not been wrong at all according to the information and taxonomic schemes present at that time. Later, Samson et al. [76] broadened the concept of *P. chrysogenum* and placed *P. notatum* in synonymy with the former, a transfer that would be later supported in several works, and thus Fleming’s penicillin-producer was identified as *P. chrysogenum* from that point on. For the whole story and a discussion on these taxonomic changes see Houbraken et al. [72], who also hypothesize on the origin of the Fleming’s strain, now *Penicillium rubens* Biourge (1923) (IMI 15378) (ATCC 8537; NRRL 824).

Working independently, another group also found clear evidence about the historical penicillin producers being a distinct species from the type species *P. chrysogenum*. Henk et al. [77] performed a phylogenetic and population genetics analysis with samples collected from air and dust in and around St Mary’s Hospital, where Fleming worked, and strains in culture collections originated from different geographical locations around the world. Genotyping of 30 markers showed that preserved fungal material from Fleming’s laboratory was nearly identical to derived strains currently in culture collections and in the same species as NRRL 1951, distinct from the *P. chrysogenum* type species. In addition, Henk et al. [77] found mating type ratios near 1:1 in the populations and evidence of recombination among loci that are physically linked, which suggested a sexual or sexual-like reproductive mode in both *P. chrysogenum* and the Fleming species (identified as *P. rubens* by Houbraken et al. [72]). As both the Fleming species (*P. rubens*) and *P. chrysogenum* show similar patterns of recombination and presence of both mating types, Henk et al. [77] concluded that they would derive from a sexually recombining ancestor, diverging around 0.8 MYA.

After the works of Hoff et al. [49], Houbraken et al. [72], and Henk et al. [77], the panorama regarding the identity and relations between the historical penicillin-producing strains radically changed. It was then made clear that *P. chrysogenum* NRRL 1951 and Fleming’s isolate, former *P. notatum* (NRRL 824), are both *Penicillium rubens*, each belonging to a different mating type in an heterothallic organization. Therefore, all strains in the lineage of NRRL 1951, which includes Wis. 54-1255 and virtually all industrial strains, historical and currently in use, are also *P. rubens*. The occurrence of sexual mating was further suggested by the presence of repeat-induced point mutation (RIP) in the genome of *P. rubens*, a process associated with meiosis [78].

### 4.3. P. chrysogenum (P. rubens) Sexual Cycle

The functionality of the mating type locus and the existence of a real sexual cycle in *P. rubens* would be finally confirmed by Böhm et al. [79]. The authors managed to induce the sexual cycle growing the fungi on oatmeal agar supplemented with biotin. Formation of cleistothecia containing ascospores was observed, as well as evidence of recombination for both molecular and phenotypic markers. One of the crosses was performed between MAT1-1 strain Q176 (obtained from NRRL 1951 in the first stages of CSI programs, see above) and MAT1-2 strain IB 08/921 (a wild isolate), and the offspring of this crossing showed novel characteristics derived from both parents, with recombined phenotypic characters such as the production of a yellow pigment (strain Q176), dark green conidia (strain IB 08/921), and penicillin production level (higher in strain Q176).

The *mat1-1-1* gene at locus MAT1-1 not only controls sexual mating in *P. rubens* but additionally has several other important functions: it positively regulates penicillin biosynthesis, partially represses conidiation (mainly in presence of light), and controls apical growth and branching in germinating conidia [79]. The functions of the *mat1-2-1* gene in strains of the MAT1-2 mating type were analyzed by Böhm et al. [50]. In addition to its role in the sexual cycle, *mat1-2-1* represses conidiation in darkness and conidia germination, and in contrast to *mat1-1-1*, it has no effect on penicillin production. In this work, the authors crossed a MAT1-1 high-producing strain (P2niaD18) with a MAT1-2 ascospore isolate (AS25) obtained from a crossing between strain Q176 (MAT1-1) and the wild isolate Pc3 (MAT1-2), resulting in the formation of cleistothecia with viable ascospores, which showed a low level (9/100) of recombination of molecular markers. This low frequency of recombination can be explained by the high karyotypic divergence between strain P2niaD18 (submitted to heavy mutagenic treatments during CSI) and strain AS25. Nevertheless, the fact that a high-producing strain like P2niaD18 retains the ability to sexually cross opens the possibility of using sexual crossing for strain improvement. Böhm et al. [50] analyzed then the genome sequence of strains Pc3, AS25, P2niaD18 (sequence previously obtained by Specht et al. [27]), and one ascospore isolate from the P2niaD18 × AS25 crossing (strain AS25-3), which showed evidence of a recombinant genotype. The results showed a rather complex pattern of recombinant sequences, reflecting recombination events in the strain AS25-3 genome that might be due to crossovers, crossover-associated gene conversions, or noncrossover gene conversions. Additionally, a mosaic-like nonrandom distribution of parental genome sequences was observed in this strain. The high levels of heterozygosity between the two mating partners, P2niaD18 and AS25, could be responsible for this recombination pattern. As a final conclusion, Böhm et al. [50] mentioned that *mat1-2-1* appears to control various developmental programs, ensuring that sexual development takes place during darkness by repressing conidiosporogenesis.

## 5. *Penicillium chrysogenum* Did It Again: Pioneering the Discovery of Gene Clustering of Fungal Secondary Metabolite Genes

If the discovery of penicillin by Alexander Fleming is often considered a serendipity (we do not fully share this view), clustering of secondary metabolite genes in fungi was the result of a more laborious process, and yet it was equally unexpected. The *pcbC* and *penDE* genes encode the enzymes catalyzing the second and third steps in penicillin biosynthesis, respectively. Both genes were cloned separately from a *P. chrysogenum* AS-P-78 genomic library constructed in the λ phage vector EMBL3 [80,81], using as probes mixtures of oligonucleotides designed from the N-terminal amino acid sequence of the previously purified enzymes isopenicillin N (IPN) synthase [82] and IPN acyltransferase [83]. The *pcbC* gene had also been cloned previously from another strain [84] using a heterologous probe from the *pcbC* gene of *Cephalosporium acremonium* [85]. It was autumn, 1988, laboratory of Microbiology at the University of León, under the direction of Professor Juan Francisco Martín. Could the *pcbC* and *penDE* genes be linked? This possibility was contemplated by José Luis Barredo and Bruno Díez, at that time working on their PhD Thesis, who decided to test it. Clones from the λ phage EMBL3 library positive for the *pcbC* gene were hybridized with a probe corresponding to the 5′end of the *penDE* gene. It was a “bingo!” moment when they saw that out of 45 *pcbC*-containing library clones, 43 gave a positive signal with the *penDE* probe. Closer examination of 10 of these clones by Southern blot and restriction mapping located both genes within a 5.1 kb SalI fragment [86]. In addition, Northern blot analysis showed that each gene was transcribed separately and thus possessed its own promoter, which indicated that was something different from a bacterial operon. The finding was unexpected: “surprisingly, the genes encoding isopenicillin N synthase and acyl-CoA:6-APA acyltransferase are linked together in a 5.1 kb SalI DNA fragment”, but the authors had the intuition that it was important: “The finding of two physically linked penicillin genes is of great interest from the evolutionary point of view and it also has relevance for improvement of industrial production of this antibiotic.”, and were they right! In parallel, they were performing chromosome walking by Northern blot to detect possible adjoining genes using probes upstream and downstream the clustered *pcbC* and *penDE* genes. Consistently, a ghostly smeary signal with an apparent huge size above 10 kb kept appearing with all probes upstream the *pcbC* gene. It was December, and Christmas was around the corner; Bruno Díez left these autoradiography films on José Luis Barredo’s desk for him to interpret the weird result as he returned from his holiday (vacation times seem to have played a role in penicillin research, with 60 years of interval). It did not take them much time (neither probably much effort) to realize that was probably the transcript of the remaining penicillin biosynthetic gene, *pcbAB*, encoding the δ-(L-α-aminoadipyl)-L-cysteinyl-D-valine (ACV) synthetase (ACVS) that catalyzes the first step of the pathway, since this was expected to be a large enzyme condensing the three amino acid precursors. The large transcript indicated that the enzyme would be a single polypeptide, which was confirmed by sequencing [87]. Working independently, Smith et al. [88,89] at the University of Bristol cloned and sequenced the *pcbAB* gene from another strain and located it together with the *pcbC* and *penDE* genes in the same cosmid clone.

The first fungal secondary metabolite gene cluster, the *P. chrysogenum* penicillin cluster, was so completed. Very shortly afterwards, clustering of the penicillin genes was also confirmed in *A. nidulans* [42,90,91]. By the mid-1990s, a half dozen additional secondary metabolite gene clusters had been identified in different species, including the trichothecene, aflatoxin, sterigmatocystin, and several melanin clusters [92]. Awareness of the huge wealth of secondary metabolites fungi can produce came with the genome sequencing of three *Aspergillus* species and the identification of multiple and distinct secondary metabolite gene clusters in their genomes [93]. From then on, the discovery of secondary metabolite clusters followed an exponential growth. Efforts for systematization of annotations for clusters, genes, and their products are underway [94], as well as the development of databases, such as BiG-FAM and IMG-ABC, offering a comprehensive and manageable layout to search for clusters and products [95,96].

The discovery that genes of secondary metabolite pathways form clusters in filamentous fungi challenged the paradigm that eukaryotic genes for related functions and metabolic pathways are usually scattered in the genome. The clustering of secondary metabolite genes has important evolutionary, functional, and regulatory implications. It greatly affected the way secondary metabolism in fungi was studied; it was now possible to use chromosome walking to clone and characterize all the biosynthetic genes of a pathway along with accessory and regulatory genes, thus facilitating the identification and characterization of the pathway and the final product. Entire clusters could now be transferred for heterologous expression in another host. Upon the arrival of the genomics era, identification of secondary metabolite gene clusters could be systematically performed with computer algorithms, and once it was found that most clusters remain silent in laboratory conditions, the clustering allowed the development of effective methods to awake silent clusters and identify the final product [97,98,99,100]. We will talk about these topics in coming sections.

## 6. Sometimes Fewer Is Better, or the Unexpected Virtue of Simplicity

In comparison to other fungal secondary metabolite gene clusters, the penicillin culster looks meagre, with just three biosynthetic genes, no regulatory genes, no transport genes, no other indispensable or important genes… nothing else relevant. And yet, the product of this cluster, penicillin, is a potent antibiotic already produced in fairly high quantities by the original wild type strain NRRL 1951 and it is today one of the most widely used drugs in medicine. In this section, we will deal with the penicillin biosynthetic genes, other genes related to penicillin production, and the absence of the latter from the amplified DNA region characteristic of high-penicillin-producing strains.

### 6.1. The Penicillin Biosynthetic Pathway: Enzymes and Genes Involved

Penicillin biosynthesis is carried out in three steps, the first two of which form the distinctive β-lactam ring responsible for the antibacterial activity. The structural core of penicillin consists of a bicycle formed by a four-membered β-lactam ring fused with a thiazolidine ring. Bound to the β-lactam ring is an acyl side-chain, the most common being phenylacetate, in the case of penicillin G (benzylpenicillin), and phenoxyacetate, in the case of penicillin V (phenoxymethylpenicillin) [101]. The β-lactam ring underlies the antibacterial activity of penicillin, while the acyl side chain determines the spectrum of activity as well as the pharmacokinetic properties of the compound [102]. The antibiotic action of penicillin G, as that of the other β-lactams, is produced by its reaction with catalytically important nucleophilic serine residues in transpeptidases (penicillin-binding proteins, PBPs) that are involved in peptidoglycan biosynthesis, thereby affecting bacterial cell-wall integrity [103].

**Figure 1:**
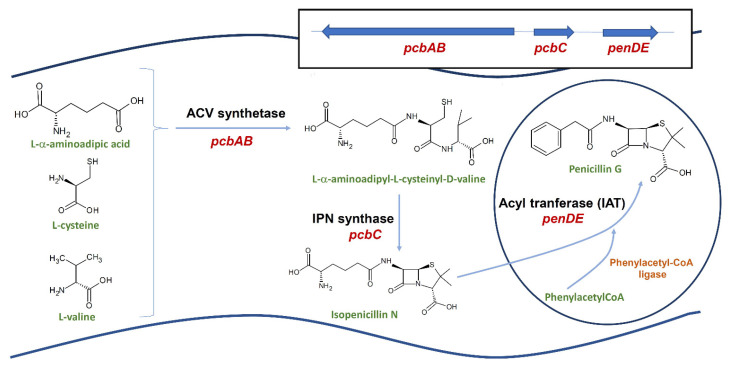
Outline of the penicillin biosynthetic pathway, showing the enzymes involved and the clustered genes encoding each enzyme.

The penicillin biosynthetic pathway has been described in detail in different review articles (e.g., [104,105,106,107]). Here, we will only make a brief description of the biochemical steps leading to penicillin biosynthesis (Figure 1). All naturally occurring penicillins are synthesized from three amino acids: L-α-amino-adipic acid (L-α-AAA), L-cysteine, and L-valine. In fungi, L-α-AAA is formed by a specific pathway related to lysine biosynthesis or by catabolic degradation of lysine [108,109]. In the first reaction of the penicillin pathway, the three amino acid precursors are condensed to form the tripeptide L-α-aminoadipyl-L-cysteinyl-D-valine, hereafter named ACV, by a non-ribosomal peptide synthetase: ACV synthetase (ACVS). The ACVS is a very large multifunctional protein with a molecular weight of 420 kDa that is encoded by the *pcbAB* gene, an intronless gene of 11 kb [87,89,110]. The ACVS has three well-conserved domains that activate each of the three amino acids with ATP, forming aminoacyl-adenylates, which are bound to the enzyme as thioesters. Next, L-valine is epimerized to D-valine, and the three amino acids are condensed to form the linear tripeptide ACV, which is released from the enzyme by the action of an internal thioesterase activity [111,112]. In the following step of the pathway, the linear tripeptide suffers an oxidative ring closure that leads to formation of the bicyclic ring. This reaction is catalyzed by the isopenicillin N synthase (IPNS), a nonheme-Fe(II)-dependent oxidase with a molecular mass of about 38 kDa [113]. The IPNS is encoded by the *pcbC* gene, with a size around 1 kb and intronless [84,105]. The resulting product, isopenicillin N (IPN), is a bioactive compound, although with weak antibiotic activity. In the final step of penicillin biosynthesis, the α-aminoadipic side-chain of IPN is exchanged for an acyl group, previously activated as acyl-CoA, to produce hydrophobic penicillins. This reaction is catalyzed by the IPN acyltransferase (IAT), a heterodimer enzyme containing two subunits: subunit α (11 kDa, corresponding to the N-terminal region) and subunit β (29 kDa, C-terminal region). The IAT is formed from a 40 kDa precursor protein (proIAT) encoded by the *penDE* gene. This gene is 1.2 kb in size and contains three introns [81,114]. Extensive biochemical characterization of IAT in *P. chrysogenum* has led to the proposal that, during penicillin formation, IPN is first converted to 6-APA, an intermediate that remains bound to one of the active site pockets. Subsequently, an acyl-CoA residue binds in another pocket of the active site, and then, 6-APA and the acyl group are linked by the enzyme, resulting in the formation of penicillin [115].

### 6.2. Ancillary Enzymes in Penicillin Biosynthesis

In addition to the core enzymes of the penicillin pathway, other accessory enzymes are required for penicillin biosynthesis, such as acyl-CoA ligases, which activate the side-chain precursor to be used in the final step of biosynthesis catalyzed by IAT [116,117], and phosphopantetheinyl transferase (PPTase), which activates the ACVS [118,119]; they will be treated in a later section about metabolic engineering. Another enzyme with an important function in penicillin production is the major facilitator superfamily (MFS) transporter PenV, located in the membrane of vacuoles, which has been proposed to participate in the formation of the ACV tripeptide through the supply of amino acids from the vacuolar lumen to the ACVS [120]. Recently, PenV has been proposed to be a stress-gated TRP channel similar to the calcium transporter CSC-type TRP of *Arabidopsis thaliana* and yeasts, with a physiological action mediated by calcium signaling in response to external stressing factors [121]. PenV was shown to increase expression of the genes *pcbC* and *penDE* [120], an effect that would be mediated by a calcium signaling cascade.

The biosynthesis of penicillin has a precise spatial organization in the fungal cell, which involves a highly structured transport system between cytosol, intracellular organelles, and the cell membrane/cell wall. The first step in penicillin biosynthesis is the formation of ACV by the ACVS. The first studies about subcellular localization of this enzyme suggested that it was attached to the vacuole membrane [122]. Later, it was confirmed that ACVS was localized in the cytosol without significant attachment to any membrane system [123]. Stand-alone PPTases are also likely located in the cytosol because of their role in the activation of ACVS and α-aminoadipate reductase [124]. After its formation in the cytosol, ACV serves as substrate of the IPN synthase. Initial experimental evidence suggested that IPNS was localized in the cytosol [123,125], probably associated with ACVS [126]. However, this co-localization hypothesis needs to be supported by protein–protein interaction studies. More recently, IPNS was located in the cytoplasm in a non-homogeneous distribution by immunodetection [127]. Particularly, IPNS seems to be associated to the endoplasmic reticulum, next to the vacuole tonoplast and to tube-like structures in the cell wall, and highly concentrated around the peroxisomes [126,127]. The location of IPNS around peroxisomes is in good agreement with the localization of IAT inside these organelles. IAT has a functional peroxisomal targeting sequence PTS1 at the C-terminal, and experimental evidence indicates that its location is inside the peroxisome [125,128,129,130]. In addition, it has been clearly shown that the peroxisomal localization of IAT is required for biosynthesis of penicillin, since the removal of the PTS1 sequence results in the mislocalization of IAT in the cytosol and lack of penicillin biosynthesis [128,131]. In agreement with this, several studies have demonstrated that proliferation of peroxisomes enhances overall IAT activity and penicillin biosynthesis [132,133]. IAT uses phenylacetyl-CoA and other CoA-activated acyl groups as a substrate. Thioester bond formation between the acyl groups and CoA is catalyzed by the phenylacetyl-CoA ligase, an enzyme also containing a PTS1 targeting sequence [116,117] and whose location in peroxisomes was confirmed by LC-MS/MS analysis of the peroxisomal protein content [30].

Taking into account that early enzymes of penicillin biosynthesis are located in the cytosol while phenylacetyl-CoA ligase and IAT are located in peroxisomes, the final step of penicillin biosynthesis requires the transport of the side chain precursors and IPN into peroxisomes. Given the hydrophilic character of IPN, the existence of an active transporter for the uptake of IPN from cytosol to peroxisomes was proposed. Fernández-Aguado et al. [134] located an MFS transporter in the *P. chrysogenum* peroxisomal membrane, named PenM, which was involved in the uptake of IPN to peroxisomes. In addition, the authors showed that PenM is not involved in the transport of phenylacetic acid (PAA). PAA is transported into the peroxisome by another MFS transporter, PaaT, which affects the levels of penicillin production when its abundance is modified in the cell [135]. Thus, two different transporters are necessary for uptake of IPN and PAA to peroxisomes.

Once produced, benzylpenicillin has to be transported out of the cell into the culture medium. For many years, significant efforts were made to find putative transporters involved in the secretion of penicillin in *P. chrysogenum* [20,124]. Several pieces of evidence suggested that secretion was an active process involving ABC transporters [126]. However, the functional analysis of a large number of ABC transporters of *P. chrysogenum* did not yield positive results [126,136]. Diffusion was also considered as a transport method; however, the accumulated extracellular levels of penicillin are much higher than the intracellular ones. Given the lack of evidence of active secretion or diffusion of penicillin, another type of transport such as macroautophagy of organelles began to be considered [137,138]. Recently, by using ultrastructural analysis by TEM and AFM, Campos et al. [139] proposed that *P. chrysogenum* exports penicillin through a vesicular transport system. In this model, multivesicular bodies are formed from the peroxisomes, which disintegrate, giving rise to a large number of vesicles. These vesicles transport the antibiotic to the vicinity of the plasma membrane and merge with it, thus releasing penicillin outside the cell.

### 6.3. Analysis of the 56.9 kb Amplified Region Containing the Penicillin Biosynthetic Genes

The *pcbAB*, *pcbC,* and *penDE* genes are clustered in a 17 kb DNA region, which is part of the 56.9 kb amplifiable DNA region (AR) that appears in multiple copies in high-producing strains [54]. This AR contains several other ORFs, most of them transcriptionally active but none essential for penicillin biosynthesis, as revealed by the capability of the *pcbAB*, *pcbC,* and *penDE* genes alone to restore full penicillin synthesis in a mutant lacking the complete region [140]. Neither are any of these genes required for normal growth and development of the fungus, as revealed by the phenotype of mutants lacking the entire 56.9 kb AR [141]. Since the number of copies of the AR is correlated to the production levels of penicillin, it should be expected that genes involved in penicillin biosynthesis other than the core biosynthetic genes were present in the AR, for instance phenylacetyl-CoA ligase, PPTase, or transport genes. However, no such genes are found in the AR [140,142]. Nevertheless, some of the genes located in the AR may provide precursors or be involved in some way in increasing penicillin production. Particularly ORF12, which encodes a protein with similarity to saccharopine dehydrogenases, may be related to biosynthesis of α-aminoadipic acid. Strain Wis. 54-1255 npe10, which lacks the entire AR and thus ORF12, shows partial lysine auxotrophy and grows deficiently in non-supplemented minimal medium [142], confirming a role of this gene in lysine biosynthesis, and lysine catabolism leads to formation of α-aminoadipic acid in *P. chrysogenum* [143]. ORF11 encodes a putative D-amino acid oxidase; these enzymes are known to convert the D-α-aminoadipic chain of cephalosporin C or penicillin N to α-ketoglutarate [144], and thus the possible involvement of the ORF11-encoded protein in deamination or isomerization of α-aminoadipic would be worth studying. ORF13 encodes a protein containing a Zn(II)2-Cys6 fungal-type DNA-binding domain, which may have a role as transcriptional regulator. However, deletion of this ORF did not cause any change in the levels of penicillin production [140]. This result confirms that the penicillin cluster contains no specific transcription factor that regulates the transcription of the three biosynthetic genes.

Finally, no synteny exists between the *P. chrysogenum* AR and the corresponding *A. nidulans* region containing the penicillin genes. These findings indicate that no functional unit related to penicillin production has been conserved between *P. chrysogenum* and *A. nidulans* other than that formed by the three biosynthetic genes. Likewise, high penicillin production in industrial strains does not seem to be dependent on the amplification of genes related to penicillin biosynthesis other than the three biosynthetic genes; for instance, the gene encoding PPTase (essential for penicillin biosynthesis) has been shown to be in single copy in both low and high-producing strains with an amplified AR [119], and no other penicillin production-related gene has been reported to be in multiple copies in industrial strains.

Thus, we can conclude that the *P. chrysogenum* penicillin gene cluster is great in its simplicity, just three genes that revolutionized medicine, biotechnology, and industrial microbiology, but where did they come from? See next section.

## 7. Where’d You Get Those Genes?

*P. chrysogenum* became famous thanks to three genes, but everything indicates that at least two of them were borrowed, and seemingly arrived in the fungus after a long and sinuous trip involving more than one step. The origin and evolution of the penicillin cluster constitute a paradigmatic example about how secondary metabolite gene clusters are formed in fungi.

### 7.1. β-Lactam Biosynthetic Genes and Their Proposed Origins

The discovery of the expansion of the penicillin N ring to deacetoxicephalosporin (DAOC) in the cephalosporin biosynthesis pathway revealed that both β-lactam compounds, penicillins and cephalosporins, were structurally related [145,146]. On the other hand, the molecular characterization of genes involved in β-lactam biosynthesis in fungi and bacteria revealed features such as high sequence similarity between prokaryotic and eukaryotic genes, their occurrence in gene clusters, and the unusual and narrow distribution of microorganisms that produce β-lactam compounds [104]. Thus, the possibility that the biosynthetic pathways of β-lactam compounds of fungi and bacteria had a common origin began to be considered, and a horizontal gene transfer (HGT) of β-lactam biosynthesis genes from bacteria to fungi was proposed by several authors [84,104,147,148,149,150].

The absence of introns in the *pcbAB* and *pcbC* genes, their high percentage of G + C content, similar to organisms such as Actinobacteria, and the phylogenetic analyses of the IPNS proteins strongly support the directionality of the transfer from bacteria to fungi [151]. However, the number of HGTs and the possibility that lateral gene transfers (LGT) between fungi also occurred during the formation of the current penicillin gene cluster is a matter of discussion. For example, Weigel et al. [147] postulated a single HGT event from *Streptomyces* to fungi, while Peñalva et al. [149] proposed that two independent HGT events occurred. According to the second hypothesis, in one event, all the genes necessary to produce a cephalosporin ring would have been transferred from *Streptomyces* to an ancestor of *Acremonium chrysogenum*, and in the other transfer event, only the ACVS and IPNS genes would have been transferred from *Streptomyces* to a penicillin producer ancestor. More recently, Martín and Liras [151] proposed a hypothesis that integrated all the evidence available to date. According to this hypothesis, there was only one HGT event of the cephalosporin biosynthetic cluster from bacteria to fungi, specifically to an ancestor of *Pochonia chlamydosporia* (order Hypocreales). To support this transfer event, the authors performed a complete phylogenetic analysis of all known IPN synthases from fungi and bacteria, which included the IPN synthases of fungi in which the presence of genes from the cephalosporin cluster has been detected (*A. chrysogenum*, *P. chlamydosporia,* and *Kallichroma tethys*, members of the order Hypocreales, and *Madurella mycetomatis* belonging to the order Sordariales, phylogenetically close to Hypocreales) and all the IPN synthases of penicillin-producing fungi (belonging to various species of *Penicillium* and *Aspergillus*, members of the order Eurotiales). The result of this analysis revealed that the IPN synthases of Hypocreales and Eurotiales diverged from a common branch of the tree, thus indicating that they probably derive from a single HGT event from bacteria to fungi. The IPN synthases of Gram-negative bacteria are closer to the IPN synthases of Hypocreales and Eurotiales fungi than those of Gram-positive bacteria, which suggests that the origin of the genes transferred to fungi was Gram-negative bacteria. This single transfer event is also in agreement with the distinct organization of the *pcbAB–pcbC* genes occurring in bacteria and fungi: same orientation in bacteria and opposite orientation in fungi. A reorganization of the *pcbAB* and *pcbC* genes could have occurred during the HGT event, maintaining afterwards the same arrangement in fungi throughout evolution. The analysis of the cephalosporin gene cluster in different cephalosporin producing fungi indicates that *P. chlamydosporia* has conserved the genes *pcbAB*, *pcbC*, and *cefEF* clustered together, while in *A. chrysogenum*, the *cefEF* gene split from the cluster to a new location in another chromosome [46]. Regarding the origin of the penicillin cluster, Martin and Liras [151] proposed that there was an LGT event of the *pcbAB* and *pcbC* genes from Hypocreales to Eurotiales after the split of these two orders, a hypothesis based on the phylogenetic distance between Hypocreales and Eurotiales and the absence of the genes specific for cephalosporin production in Eurotiales.

However, orthologs of *cefD1* and *cefD2*, responsible for the epimerization step converting IPN into penicillin N in cephalosporin-producing fungi, are present in *P. notatum* (original Fleming isolate), as revealed by the sequencing of its genome [24], as well as in *P. chrysogenum* strains used in CSI and industry [24,30]. The *cefD1* and *cefD2* genes are expressed, and their respective protein products localize to microbodies (functionally peroxisomes) in the industrial strain *P. chrysogenum* DS17690 [30], which is also the location of these two proteins in *Acremonium chrysogenum* [126]. Since IPN is transported into peroxisomes to serve as a substrate for the last step of penicillin biosynthesis, there exists the possibility that penicillin N is also synthesized in *P. chrysogenum*, although, to our knowledge, there are no reports on this. In *A. chrysogenum*, penicillin N serves as a substrate for the enzyme that catalyzes the biosynthesis of deacetyl cephalosporin (DAC), which is encoded by the *cefEF* gene of prokaryotic origin, a gene that is absent in *P. chrysogenum*, thus precluding the possibility that *P. chrysogenum* can synthesize cephalosporin. In addition, penicillin N could not be used as a substrate of the IAT in an ^35^S isotopic exchange assay between penicillins and 6-amino-penicillanic acid (6-APA) [152], which would imply that, if produced, penicillin N would probably accumulate in the peroxisome rather than be used for penicillin G or V biosynthesis.

Another gene worth studying that can shed light on the HGT/LGT events that led to the formation of the penicillin cluster is *cefT*, which encodes an MFS transporter located in the plasma membrane of *A. chrysogenum* and has a role in export of cephalosporin C (CPC) [126] and other cephem intermediates of the CPC pathway to the medium [153], although other redundant systems must exist to transport the antibiotic out from the cell. While CefP, which transports IPN into the peroxisome in *A. chrysogenum*, has no strict ortholog in *P. chrysogenum* [120], CefT is highly similar (65.7% identity head-to-tail) to, and thus probably an ortholog of, *P. chrysogenum* PaaT, which transports PAA into the peroxisome to be used as a substrate of the IAT [135]. PaaT contains 12 transmembrane domains and one Pex19p-binding domain for Pex19-mediated targeting to the peroxisome membrane. PaaT orthologs are also present in *A. nidulans*, *A. oryzae* (both penicillin producers), and *Aspergillus clavatus* and other fungi that are not known to produce penicillin (Martín [126] and references therein).

### 7.2. A Possible Pathway for the Formation of the Penicillin Gene Cluster

**Figure 2:**
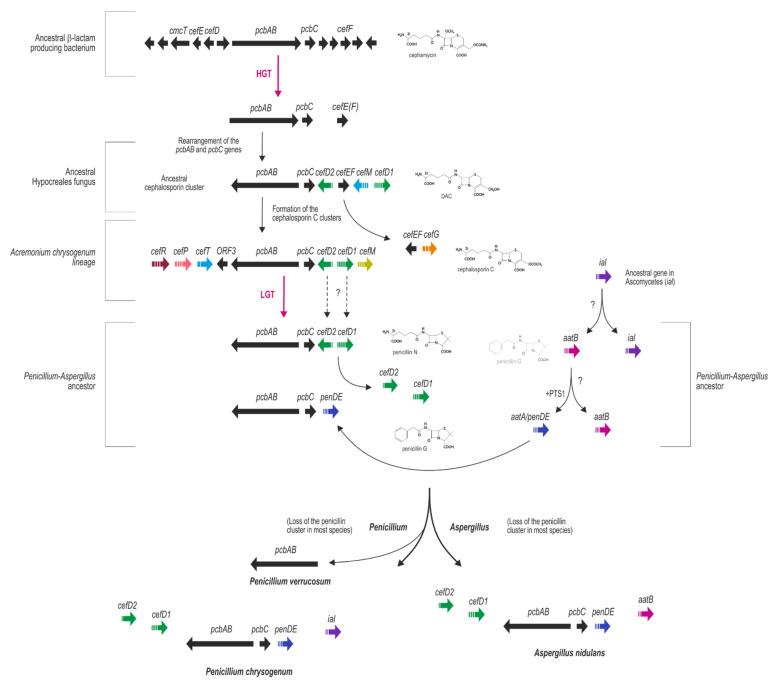
Hypothetic pathway for the formation of the cephalosporin and penicillin gene clusters, which includes one HGT from bacteria to Hypocreales and one LGT from a Hypocreales fungus to an ancestor of *Penicillium* and *Aspergillus* (see text for more details). Genes in black are of bacterial origin, and genes in different colors are of eukaryotic origin. Introns are indicated with a white bar. At the right of each cluster is the name and structure of the β-lactam antibiotic that it produces (for old, hypothetical clusters no longer present in current species the antibiotic might have had a different structure). The grey color of penicillin G beside the *aatB* gene indicates low production of this antibiotic due to the low IAT activity of the cytosolic AatB enzyme. DAC: deacetyl cephalosporin. PTS1: peroxisome targeting sequence type 1.

Several questions remain open regarding the transfer of β-lactam genes from bacteria to fungi and the formation of the cephalosporin and penicillin clusters: how many genes were transferred from bacteria to fungi? Was there a single or more HGT events? Was there an LGT from Hypocreales to Eurotiales? And if so, how many genes did it involve? Finally, how was the final penicillin gene cluster formed? Here, we present a hypothesis, based mainly on the proposed by Martin and Liras [151], including some additional possibilities that are yet to be proven and that can serve as an outline for further studies on this topic. Alongside, we propose some properties that would have been acquired by the recipients upon transferring of the genes and during the evolution of the clusters. Figure 2 summarizes the main events in the formation of the eukaryotic β-lactam gene clusters.

1. An HGT event involving at least the genes *pcbAB*, *pcbC,* and *cefE* or *cefF* occurred from bacteria to an ancestor of the currently cephalosporin producing fungi, which belong to the orders Hypocreales and Sordariales. This is supported mainly by the comparison of IPNS sequences [151] and the conservation of the arrangement of the genes *pcbAB* and *pcbC* in all β-lactam-producing fungi. The transferred genes are sufficient to synthesize DAC provided a mechanism to isomerize IPN into penicillin N is available (see point 2). *cefE* and *cefF* are believed to have originated in bacteria after gene duplication of an ancestral gene whose product possessed both penicillin N expandase and DAOC hydroxylase activities; then, CefE and CefF would have evolved to specialize in each of the activities. Thus, the gene transferred to fungi might have been an ancestor of *cefE*/*cefF* before duplication.
2. The function of IPN epimerization to penicillin N was developed de novo in the cephalosporin-producing ancestor. The epimerization system is different in bacteria and fungi, involving only one step in bacteria, catalyzed by the product of the *cefD* gene, and three steps in fungi: isopenicillinyl N-CoA synthetase (catalyzed by the product of the *cefD1* gene), racemase (catalyzed by the product of *cefD2*), and thioesterase. Although the bacterial *cefD* might have been transferred along with the other genes it resulted not functional in fungi. Alternatively, the *cefD* gene was not transferred. Whatever the case, fungi evolved a system to isomerize IPN (with an L-AAA side chain) to penicillin N (with a D-AAA side chain). The *cefD1* and *cefD2* are clearly of eukaryotic origin, they both contain introns, and they must have been recruited to the original cluster transferred from bacteria early in the evolution, since all characterized cephalosporin producers have these two genes clustered with genes *pcbAB*, *pcbC*, and (with the exception of *A. chrysogenum*) *cefEF*. CefD1 is highly similar to very-long-chain-fatty acid-CoA synthetases, whereas CefD2 is highly similar to human racemases [154]. Members of both families have been found in mammalian microbodies [155]. We can speculate that the cephalosporin-producing ancestor evolved a transport system (the CefP transporter) to introduce IPN into the peroxisome, where it would be converted to penicillin N by enzymes residing there. The penicillin N would then be used as the substrate of the expandase/hydroxylase enzyme (product of the *cefEF* gene) for DAC formation.
3. The *cefG* gene, of eukaryotic origin and encoding the last enzyme of CPC biosynthesis [156], is present in *A. chrysogenum* but absent in other cephalosporin producers such as *Pochonia chlamydosporia* and *Madurella mycetomatis*. This indicates that this gene evolved later, after the split of *A. chrysogenum* from the other species. Another difference between *A. chrysogenum* and the other species is that, while in the latter the *cefEF* gene has been maintained in its original location clustered with the *pcbAB–pcbC* genes, in *A. chrysogenum* it moved to a new location in another chromosome. Most likely, the *cefG* gene was recruited to this new location to form the so-called “late” cephalosporin cluster [46,156].
4. An LGT event, involving at least the *pcbAB* and *pcbC* genes, occurred between Hypocreales and an ancestor of *Penicillium* and *Aspergillus* (Eurotiales). There exists the possibility that the *cefD1* and *cefD2* genes were transferred alongside *pcbAB* and *pcbC*. We have performed Blast searches of *A. chrysogenum* CefD1 and CefD2 in the kingdom Fungi, and the results suggest that the *cefD1* and *cefD2* genes may have been transferred to a *Penicillium*-*Aspergillus* ancestor by LGT. CefD1 appears in cephalosporin producers and other Hypocreales (*Claviceps*, *Metarhyzium*, *Torrubiella*), with identities of 60–65% in the case of *Claviceps* spp., and next in many penicillin-producing and non-producing species of the genera *Penicillium* and *Aspergillus*, with identities of 45–50%, but not in other Eurotiales or members of other taxa. Regarding CefD2, it appears in cephalosporin producers but not in other Hypocreales, and next in many *Penicillium* and *Aspergillus* species (penicillin-producing and non-producing), with high identity percentages of around 65%, but not in other Eurotiales or other taxa. The *cefD2* genes in *A. chrysogenum* and *P. chrysogenum* contain a single intron whose position is conserved at the beginning of the ORF. This transfer would have endowed the *Penicillium*-*Aspergillus* ancestor with the capacity to produce penicillin N, which possesses some more antibacterial activity than IPN [157]. Eventually, the *penDE* gene (see below) joined the imported penicillin N cluster, which meant a dramatic improvement in antibiotic capacity owing to the much stronger antibacterial activity of penicillins G or V with respect to IPN or penicillin N. We can speculate that conserving together the *pcbAB*, *pcbC,* and *penDE* genes was advantageous for the fungus, while the *cefD1* and *cefD2* genes were now much less important, or even detrimental regarding antibiotic production and thus they were not conserved in the new penicillin cluster. However, these genes were not lost either, and they continue being transcribed and targeted to peroxisomes [30]. The *cefT* gene might also have been transferred with the other genes from Hypocreales to a *Penicillium*-*Aspergillus* ancestor. *A. chrysogenum* CefT and *P. chrysogenum* PaaT are highly similar (65.7% identity head-to-tail) and share some functional characteristics, such as the capacity to transport PAA [158,159], which is the function that PaaT performs in penicillin biosynthesis, tranporting PAA into the peroxisome [135].
5. The *penDE* gene, of eukaryotic origin, was recruited to the *pcbAB–pcbC* cluster to eventually form the definitive penicillin biosynthetic cluster. During the process, the *penDE* gene acquired a peroxisome targeting sequence (PTS1). Below, we will describe the origin of the *penDE* gene in more detail.
6. After the split of the genera *Penicillium* and *Aspergillus*, the penicillin gene cluster was inherited by both containing just the *pcbAB–pcbC*-*penDE* genes, in the same arrangement and with several regulatory elements already present in the gene promoters (see next section), with no other relevant genes for penicillin biosynthesis in the vicinity of the cluster (20–25 kb upstream and downstream).
7. Most of the *Penicillium* and *Aspergillus* species lost the cluster, all genes at once or in more than one step, as observed in *P. verrucosum*, which contains the *pcbAB* gene but lacks *pcbC* and *penDE* [160]. The adaptation to different environments and ecological niches must have played a role in this process, but not enough data and studies are available to postulate particular environments that may favor or act against the presence of the penicillin cluster. In a study conducted by Prigent et al. [161], aiming to find correspondences between phylogenetic clades, habitats and metabolic clades elaborated from genome-scale metabolic models of 24 *Penicillium* species, two of the penicillin producers (*P. rubens* and *P. flavigenum*) were assigned the same habitat, desert plants, while the other two were included in other habitats. Nevertheless, many *Penicillium* species can be found in a wide range of habitats.
8. In a lineage leading to *P. chrysogenum* NRRL1951, a decrease in PAA catabolism by a mutation in the *pahA* gene led to an important increase in penicillin production capacity, while the penicillin cluster became placed within an amplifiable DNA region of 105.2 kb. No amplification of the penicillin cluster seems to have occurred naturally, but the stage was set for amplification to occur once strain NRRL 1951 was picked out for penicillin production and CSI programs started to be developed (shifting the amplifiable region from 105.2 to 56.9 kb in most cases).

### 7.3. Origin of the penDE Gene

The *penDE* gene has a eukaryotic origin. It contains three introns, and several features of this gene indicate that it was formed by fusion of two eukaryotic DNA fragments, one of them containing introns [162]. No known close homologues to *penDE* (*aatA* in *A. nidulans*) have been identified outside the group of penicillin producers, but a putative paralogue of the *aatA* gene was identified in *A. nidulans*, which was named *aatB* [163]. In *P. chrysogenum*, a putative *penDE* paralogue was also identified, named *ial*, which is not homologous to *aatB* [164]. *A. nidulans aatB* also contains three introns but lacks a peroxisomal targeting sequence and is located in the cytosol. The *aatB*-encoded protein possesses some IAT activity and is able to partially restore penicillin production in an *aatA*-disrupted mutant [163], while the *ial* product does not show IAT activity [164]. In addition, both *aatA* and *aatB* are regulated by the same transcription factors, AnCF and AnBH1, by binding to their respective promoters [163]. These findings led the authors to conclude that *aatA* and *aatB* are paralogues, and one of them, *aatA*, was recruited to the penicillin gene cluster of *A. nidulans*. In silico analyses of the genomes of several Aspergilli and other ascomycetes revealed the presence of genes homologous to *aatA* (*penDE*), *aatB,* and *ial* [115,164]. All of them conserve the essential motif for autocatalytic cleavage of IAT, suggesting that they are active NTN hydrolases. *Ial* gene homologues are found in *Aspergillus* spp. And *Penicillium* spp. Producing and non-producing penicillin and in several other distantly related ascomycetes, and they form a separate phylogenetic clade, whereas *penDE* (*aatA*) homologues have been found only in penicillin-producing fungi (e.g., *P. chrysogenum*, *A. nidulans*, and *A. oryzae*). Regarding *aatB*, only one homologue was identified in the non-penicillin producer *A. terreus* by García-Estrada et al. [164]. On the other hand, *ial* homologues contain less than three introns (with one exception), while *penDE*/*aatA* homologues, plus *aatB*, contain three [164]. Taking into account all these data several alternative hypotheses can be proposed. One of these hypotheses, suggested by García-Estrada et al. [165], is that the *ial* gene has a different origin from the IAT-encoding genes (*aatA*/*penDE* and *aatB*), and only *aatB* would be a real paralogue of *aatA*/*penDE*. This hypothesis involves the possibility of the penicillin cluster having been formed only in the *A. nidulans* lineage (which would have been then the only receptor of the *pcbAB–pcbC* genes from Hypocreales) and transferred from there to an ancestor of the phylogenetically close *A. flavus* and *A. oryzae* and to an ancestor of the phylogenetically related penicillin-producing *Penicillium* species, which includes *P. chrysogenum* (the three species containing an *ial* homologue while lacking an *aatB* homologue). Analyzing the data and after performing some Blast searches, we propose an alternative, yet speculative, scenario in which a first duplication of the ancestral *ial* gene in the lineage leading to *Penicillium*-*Aspergillus* gave rise to the *aatB* gene, whose product would have acquired IAT activity after the transfer of the *pcbAB–pcbC* cluster from Hypocreales. In such a case, low amounts of penicillin G could be produced in the cytosol. A second duplication, in this case of the *aatB* gene, would give rise to the paralogue *aatA* (*penDE*) gene, which would eventually have been recruited to the *pcbAB–pcbC* cluster, acquiring a PTS1 peroxisome-targeting sequence in the process. After the split of *Penicillium* and *Aspergillus* and during speciation, the *ial* gene would have been lost in *A. nidulans*, while retained in other species (including the penicillin producers *A. oryzae* and *A. flavus*) as well as in *P. chrysogenum* and other species of this genus. Conversely, the *aatB* gene would have been lost in most *Aspergillus* species (and in *P. chrysogenum*) and retained in *A. nidulans* and *A. terreus*. In support of this scenario are the findings of Gidijala et al. [115], who found that the genome region around the *ial* gene in *P. chrysogenum* conserves synteny in *A. nidulans* and *A. terreus*, but an *ial* ortholog is only present in *A. terreus*, while in *A. nidulans*, a DNA region containing the *ial* ortholog seems to have been deleted during evolution. On the other hand, the genomes of *A. oryzae* and *A. flavus* contain *aatB* pseudogenes comprising only the first three exons of the gene [115] while both contain *ial* orthologues. Perhaps, *ial* and *aatB* have redundant functions (they would have originated from a common ancestor) and thus only the presence of one of them is required, as happens in all species analyzed by García-Estrada et al. [164] and Gidijala et al. [115] with the exception of *A. terreus*.

### 7.4. Concluding Remarks on the Origin and Distribution of β-Lactam Genes

HGT between bacteria and fungi and LGT between phylogenetically distant fungi seem to be frequent phenomena, and particularly common regarding secondary metabolite clusters and genes (Martín and Liras [151] and references therein). Identification of the exact routes that genes have followed throughout evolution and how they have become arranged in currently observed clusters is often a complex puzzle. Thus, the hypotheses we can propose are just that, hypotheses, and definitive answers will have to wait for more data to be available and more studies to be performed.

Finally, it is very interesting to note that the β-lactam biosynthetic cluster was not only transferred from bacteria to fungi. In recent years, the presence of genes encoding IPNS and ACVS and two cephamycin C genes (*cmcI* and *cmcJ*) has been observed throughout the springtail (Collembola, Hexapoda) clade [166,167], suggesting an HGT from a bacterial donor. Although the analysis carried out to date on different animal phyla have not found penicillin genes in them [167], it cannot be ruled out that they are present in other animal taxa through other HGT events.

## 8. An Orchestra without a Director

Secondary metabolite gene clusters are most often arranged as an ensemble, with all the necessary elements to perform their role (the biosynthesis of a compound), which includes a gene or set of basic genes encoding the enzymes forming the backbone, genes to modify this basal structure and shape the final product, accessory genes such as those to transport the product outside the cell, and a director for all these functions to be performed coordinately: a regulatory gene (eventually two) responsible for activation of expression of all or most of the genes in the cluster. PatL and CalC in the patulin and calbistrin biosynthetic clusters, respectively, are examples of this, among many others [168,169,170]. However, no such specific regulatory genes are present at or near the penicillin gene cluster [140,142], and hence regulation of the penicillin genes is dependent on general, wide-domain, transcription factors that respond to different environmental and cellular cues [171,172,173].

The absence of specific regulatory genes in the penicillin gene cluster is usually taken as evidence for the hypothesis of HGT of the β-lactam genes from bacteria to fungi; bacterial regulatory genes might have been transferred along with the biosynthetic genes *pcbAB* and *pcbC* (plus *cefEF* of *A. chrysogenum*) and lost afterwards not being functional in fungi. Alternatively, only parts of the bacterial clusters, containing the biosynthetic genes, would have been transferred. Once the biosynthetic genes were in place and functional in fungi, general (wide domain) transcription factors would have been recruited by the evolutionary appearance of cis-acting regulatory elements in the promoters of the genes. The intergenic *pcbAB–pcbC* region and the *penDE* gene promoter show a high concentration of putative regulatory sequences for binding of different transcription factors [173], far above random occurrence of these sequences, which implies strong selective pressure for the appearance of regulatory elements that allow transcriptional regulation of the genes. In this respect, it is interesting to note that the only gene of eukaryotic origin in the penicillin cluster, *penDE*/*aatA*, is a putative paralogue of another gene, *aatB* (see previous section), and both share common regulatory elements [163]. It has been shown that in gene duplication events in *Saccharomyces cerevisiae*, the regulatory elements tend to be co-duplicated with the genes [174]. A similar situation occurs in *A. nidulans*; Brakhage et al. [172] proposed that, after duplication, recruiting of the *aatA* gene to the penicillin cluster also involved the recruiting of transcriptional factors to bind to regulatory elements in the *aatA* gene promoter, later extending their function to the *acvA* and *ipnA* (*pcbAB* and *pcbC* homologues) gene promoters. This hypothesis is based on the ability of the transcriptional regulators AnCF and AnBH1 to bind in vitro to the *aatA* and *aatB* gene promoters and to regulate their expression in vivo [163], while AnCF also binds CCAAT boxes in the *acvA*-*ipnA* intergenic region and regulates expression of the *ipnA* gene [175]. Similar events might have taken place in *P. chrysogenum*. Regulatory elements for the same transcription factors are usually present in both the *penDE* gene promoter and the *pcbAB–pcbC* intergenic region, often in multiple copies. These data suggest that there is selective pressure for coordinated expression of the three penicillin biosynthetic genes.

**Figure 3:**
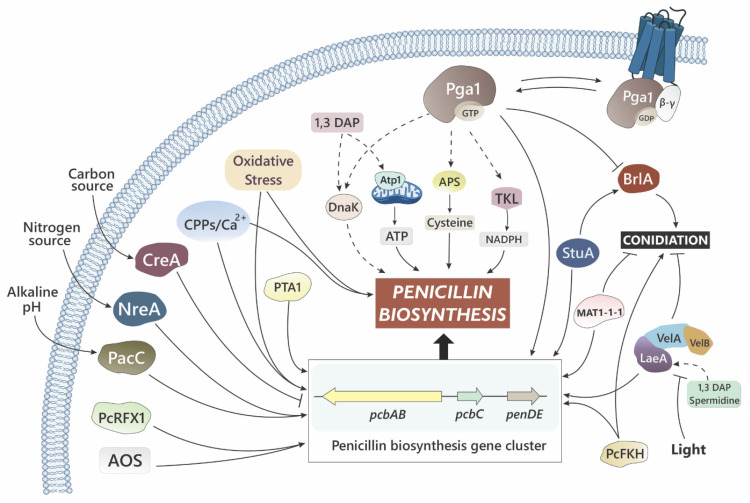
Outline of regulatory signals, transduction pathways, and proteins controlling expression of penicillin genes and penicillin production in *P. chrysogenum*.

Next, we summarize the main regulatory signals, transduction pathways and proteins controlling expression of the penicillin genes and penicillin production that have been so far identified (Figure 3).

### 8.1. Regulatory Signals and Transcription Factors

Nutritional signals were among the first to be discovered that regulate penicillin biosynthesis (Brakhage [105] and references therein). Glucose and other assimilable sugars can suppress secondary metabolite production in fungi, and the biosynthesis of penicillin is a classical example. Penicillin biosynthesis is strongly regulated by glucose but not by lactose [105,176]. In a study using suppression subtractive hybridization, the expression patterns of some of the genes supported the hypothesis that glucose induces higher respiration rates while repressing secondary metabolism [177]. Cepeda-García et al. [178] showed that the repressive effect exerted by glucose on penicillin biosynthesis is mediated by CreA, a Cys2-His2-type zinc finger transcription factor. Full derepression of penicillin biosynthesis was observed when *creA*-knocked down strains were grown in glucose-containing medium. There are six consensus CreA-binding sites (CreA1-CreA6) in the *pcbAB–pcbC* intergenic region of *P. chrysogenum*. The CreA-1 site, the most proximal to the *pcbAB* gene, plays a very important role in glucose repression, as determined by mutating this site in a P*pcbAB*::*lacZ* reporter gene fusion [178]. The importance of CreA-binding sites in the regulation of penicillin biosynthetic gene expression is supported by the findings described by van den Berg [36].

Another nutritional signal that exerts strong regulation on penicillin biosynthesis is the nitrogen source. By using gene fusions of the *pcbAB* and *pcbC* gene promoters with the *E. coli* reporter gene *uidA*, Feng et al. [179] showed that promoter activities were repressed by the addition of 40 mM ammonium to mycelium grown in the presence of lactose. In *P. chrysogenum*, the nitrogen catabolite repression is mediated by GATA transcription factors called NREA and B [180]. NREA binds to the *pcbAB–pcbC* intergenic region in response to the nitrogen source, which suggests that it mediates the nitrogen source regulation of penicillin biosynthesis [181].

The pH also affects penicillin biosynthesis. Production is higher at alkaline pH, and this regulation is mediated by PacC, a transcription factor of the Cys2His2-type [182]. *pacC* transcript levels are higher under alkaline than under acidic growth conditions and the transcript levels of the *pcbC* gene follow the same pattern, suggesting that *pcbC* is under PacC control [183]. The activity of the *pcbAB* gene promoter is also under pH control; in a reporter gene analysis, Chu et al. [183] found a higher β-galactosidase activity at alkaline pH. A total of seven PacC consensus binding sites are present in the *pcbAB–pcbC* intergenic region, six of which were bound by a purified GST::PacC fusion protein [182], and three more in the promoter of the *penDE* gene, thus confirming the trend to a high concentration of regulatory elements in the promoters of the penicillin biosynthetic genes, as also occurs with CreA consensus sites. Taken together, the above suggests that optimal growing conditions (nutrient sources, pH) lead to reduced penicillin production in *P. chrysogenum*.

Several studies have reported an association between response to oxidative stress and secondary metabolism in filamentous fungi [184,185,186,187]. Recently, it has been shown that penicillin biosynthesis is also subject to oxidative stress regulation in *P. chrysogenum*. When intracellular reactive oxygen species (ROS) were increased by the addition of H_2_O_2_ to the culture, proportional increments in penicillin biosynthesis were obtained, whereas addition of N-acetyl-L-cysteine (NAC), which decreases ROS concentration, caused a reduction of penicillin production [188].

A sequential deletion of the *pcbAB* gene promoter allowed the identification of a region that is important for promoter activity [189]. When this region was incubated with a protein crude extract, a specific and defined retardation complex could be observed in an EMSA. By using an UIA (Uracil Interference Assay), the palindromic heptanucleotide TTAGTAA was identified as the binding site for the presumed transcription factor, which was named PTA1 (Penicillin Transcriptional Activator 1). Mutations of this sequence in a P*pcbAB*::*lacZ* reporter gene fusion confirmed that this sequence is required for high levels of promoter activity [189]. Recently, a Yap1 ortholog has been characterized in *P. chrysogenum*, the bZIP transcription factor PcYap1, which seems to correspond to the PTA1 transcription factor [190]; these results link the role of the TTAGTAA regulatory sequence to oxidative stress control of penicillin biosynthesis.

Winged helix regulators are a set of evolutionarily conserved transcription factors formed by members of the RFX and the forkhead (FKH) families [191]. One transcription factor of each type has been shown to regulate transcription of the penicillin biosynthetic genes. Domínguez-Santos et al. [192] characterized the first member of the RFX family in *P. chrysogenum*, which was named PcRFX1. The authors observed that knocking down of *Pcrfx1* reduced IPN and penicillin G production as a consequence of a decreased expression of the complete penicillin gene cluster, while mutations of the PcRFX binding sites in the promoters of the three penicillin genes reduced promoter activities in fusions with the *lacZ* reporter gene [192]. For its part, the PcFKH1 forkhead transcription factor was also shown to participate in the regulation of penicillin production [193]. PcFKH1 positively controls penicillin biosynthesis through the interaction with specific binding sites in the promoter region of the *penDE* gene, inducing its transcription.

As mentioned above, Böhm et al. [79] found that the *mat1-1-1* gene, which determines the sexual mating type MAT1-1 in classical penicillin-producing strains, is involved in the regulation of penicillin production. ∆*mat1-1-1* mutants showed a significant reduction in penicillin production compared with control strains, as well as an important decrease in the transcription of the three penicillin biosynthetic genes [79]. Another developmental regulator, the transcription factor StuA, also participates in secondary metabolism regulation in filamentous fungi [194,195,196,197]. In *P. chrysogenum*, inactivation of StuA downregulated expression of six putative secondary metabolite clusters, among which the penicillin and roquefortine C clusters were affected most significantly, causing a strong reduction in Penicillin V production [198]. Therefore, both StuA and MAT1-1-1 are part of the complex network that links differentiation with secondary metabolism in filamentous fungi.

### 8.2. The Heterotrimeric G-Protein Signal Transduction Pathway

The heterotrimeric G-protein signaling pathway is one of the most important and most widely studied in fungi [199,200,201]. This signaling pathway regulates processes as diverse as cell growth and division, mating, morphogenesis, virulence establishment, and secondary metabolite production [199,201,202,203,204,205]. In *P. chrysogenum*, the class I Gα subunit, named Pga1, was identified and characterized [206]. Pga1 plays a central role in the regulation of the whole growth-developmental program of *P. chrysogenum* [207,208]. In addition, Pga1-mediated signaling regulates secondary metabolism, increasing the production of penicillin, the yellow pigment chrysogenin and the mycotoxin roquefortine [209]. In the case of penicillin, upregulation is exerted by controlling expression of the penicillin biosynthetic genes; constitutive activation of Pga1 increased the expression of the *pcbAB*, *pcbC*, and *penDE* genes, whereas both the deletion of the *pga1* gene and the mutational inactivation of Pga1 caused a decrease of the transcript levels [209]. Therefore, Pga1-mediated signaling is one of the pathways by which secondary metabolism is linked to developmental processes in *P. chrysogenum*.

According to a metabolome study of a high-yield industrial strain in penicillin-producing and non-producing conditions [29] and to a proteomics study of low, intermediate, and high-yield penicillin-producing strains [31], the main features of primary metabolism that have a major impact on penicillin production are: (i) high cysteine, but not valine or α-aminoadipate, availability; (ii) high NADPH supply; and (iii) high ATP supply. Recently, a comparative proteomic study of *P. chrysogenum* mutants with different levels of activity of the Pga1-mediated signaling pathway showed that Pga1 has a role in all these processes, stimulating the expression of proteins involved in the synthesis of NADPH (Transketolase of the pentose phosphate pathway), ATP (F1-ATPase alpha-subunit Atp1), and cysteine (sulphate adenylyl transferase) [210]. Other differentially expressed protein that may be related to penicillin biosynthesis was a dnaK-type chaperone. This protein is less abundant in the Δ*pga1* strain, which produces lower penicillin amounts. Notably, the changes found in the proteomic analysis of the Pga1-mediated signaling pathway [210] agree with results observed in other proteomics studies associated to strains or conditions of high penicillin production [31,33,38], thus confirming the role of the Pga1 pathway as an activator of penicillin biosynthesis.

### 8.3. The Velvet Complex

In filamentous fungi, the velvet family of regulatory proteins plays a key role in coordinating secondary metabolism and differentiation processes [60]. The most widely studied proteins are included in the heterotrimeric Velvet complex, which results from the interaction between VeA (VelA), VelB, and LaeA [211,212]. Bayram et al. [211] proposed a light-dependent mechanism by which the Velvet complex is formed. In darkness, the VelB-VeA dimer enters the nucleus, where it binds to LaeA to regulate expression of different genes. The effect of the Velvet complex on the secondary metabolism is mainly based on the role of LaeA. The laeA (loss of *aflR* expression A) gene encodes a putative SAM-dependent methyltransferase that acts as a master regulator of secondary metabolism in filamentous fungi [213,214,215,216,217]. In *P. chrysogenum*, overexpression of the *laeA* gene (*PclaeA*) resulted in a 4-fold increase in the transcript levels of the *pcbC* and *penDE* genes, which led to increased penicillin production [216,218]. Conversely, *PclaeA* knock-down mutants exhibited reduced levels of expression of the penicillin genes and antibiotic production. In addition, *PclaeA* knock-down caused failures in conidiophore development in both light and dark conditions [218,219].

LaeA is not the only member of the Velvet complex that affects the production of secondary metabolites. In *P. chrysogenum*, PcVelA has been shown to activate transcription of the penicillin biosynthetic gene cluster [219]. Interestingly, PcVelA is involved in the control of *PclaeA* expression, whose role in penicillin biosynthesis is also positive. Moreover, transcriptome analysis of *PclaeA* and *PcvelA* deletions in strain P2niaD revealed a widespread impact, with over 10% of the genome being affected by these deletions, with a clear overrepresentation of genes involved in secondary metabolite pathways and in fungal development [219]. These findings indicate that PcVelA and PcLaeA affect both secondary metabolism and developmental processes in *P. chrysogenum*. Recently, a comparative transcriptomic analysis of wild-type and production strains of *A. chrysogenum* and *P. chrysogenum* showed that the expression of Velvet complex target genes was altered as a consequence of strain improvement programs [35]. Furthermore, it was found that the Velvet complex controls the expression of about 50% of all secondary metabolite clusters in both fungi.

### 8.4. Exogenous Inducers

Casein phosphopeptides (CPPs) containing chelated calcium increase the secretion of extracellular homologous and heterologous proteins in filamentous fungi [220,221]. Recently, the synergistic effect of Ca^2+^ and CPPs on penicillin production in *P. chrysogenum* was reported [222]. Simultaneous addition of CPPs and CaCl_2_ greatly promotes expression of the three penicillin biosynthetic genes, increasing penicillin production up to 10– 12-fold. On the other hand, the use of alginate oligosaccharides (AOS) enhanced the production of penicillin in different *P. chrysogenum* strains [223,224,225], an effect caused by upregulation of the penicillin biosynthetic genes [225]. Transcriptional activation of the penicillin biosynthetic genes by AOS was later corroborated by Nair et al. [226].

### 8.5. Endogenous Inducers

Martín et al. [227] isolated from culture broths a molecule with autoinductive activity, which was identified as 1,3-diaminopropane (1,3-DAP). 1,3-DAP increased penicillin production by stimulating transcription of the biosynthetic genes *pcbAB*, *pcbC*, and *penDE*, an effect that was reproduced by spermidine but not by other polyamines. This stimulatory effect is exerted, at least in part, through an increase in the expression of the *laeA* gene [228]. A similar LaeA-mediated effect of 1,3-diaminopropane and spermidine was also observed in the production of lovastatin by *A. terreus* [229]. In addition, CPC production in *A. chrysogenum* is likewise increased by these two polyamines concomitant with the upregulation of the biosynthetic genes [230]. Addition of 1,3-DAP or spermidine to *P. chrysogenum* cultures also stimulates the synthesis of enzymes involved in the biosynthesis of penicillin precursors [33]. Interestingly, 1,3-DAP causes an increase in the abundance of the Atp1 and the dnaK-type chaperone, two proteins that were found decreased in the Δ*pga1* strain (see above), which produces lower penicillin amounts [210].

## 9. Treasure Island

According to calculations made by Nancy Keller [231] on previously published data, considering just two of the genera with high secondary metabolite production capacity, *Penicillium* and *Aspergillus*, with 354 and 339 currently known species, respectively, and assuming an average of 50 biosynthetic gene clusters (BGCs) per species, we would get 34,650 BGCs, which should be corrected downward to around 25,000 if the duplication rate were 25%. Adding genera from other metabolite-rich classes, the number could grow to up to 500,000, and if we include additional BGC-rich ascomycete taxa and basidiomycetes, the numbers would rise to several million. A true treasure island of new compounds with the most diverse biological activities awaiting to be explored and eventually put to use.

### 9.1. BGCs in P. chrysogenum Wis. 54-1255 and Their Connexions to Other Fungi

The genus *Penicillium* is a lavish producer of secondary metabolites. In a study performed by Nielsen et al. [232], who sequenced the genomes of nine species and analyzed in addition 15 previously published genomes, a total of 1317 putative BGCs were identified, which gives an average of 55 BGCs per genome. Analysis of the pangenome of these species revealed that 3249 gene families were shared by all of them, which represents the core genome, while 8784 gene families were present in a subset of the species, representing the dispensable genome. In the pangenome, secondary metabolism showed the greatest variation in gene content, which is expected; however, unexpectedly, genes categorized as “secondary metabolism” are mainly part of the core genome. In addition, analysis of gene copy number relative to genome size showed that secondary metabolism genes were enriched among gene families correlating with genome size. These findings indicate that the secondary metabolism gene subsystem is under positive selection and contributes to the genome expansion in the Penicilli, highlighting the importance of secondary metabolism for this genus. Nevertheless, although pooled individual secondary metabolite genes lie mainly within the core genome, the number of BGCs that is shared by many species is low, and only three BGCs are present in all 24 species analyzed.

*P. chrysogenum* Wis. 54-1255 was one of the strains whose genome was analyzed by Nielsen et al. [232]. The penicillin gene cluster is present in only three phylogenetically related species of the 24 analyzed: *P. chrysogenum*, *P. nalgiovense,* and *P. griseofulvum* (previously reported also by Laich et al. [233] and [160]). In another study performed by Prigent et al. [161] using the same genomic information as Nielsen et al. [232], metabolic clades were established based on an automatic reconstruction approach of the metabolic network of each species. Comparing the phylogenetic clades with the metabolic clades and the preferred habitat for each species, the authors concluded that often phylogenetically close species sharing a common habitat do not cluster together in a metabolic clade. However, the three penicillin-producing species mentioned above constitute one of the defined metabolic clades but have distinct habitats. Nevertheless, species can occupy different habitats, and the three penicillin producers are often found together growing on meat products such as smoked-dried meat (ham or cecina) or cheese [160,234]. These data are insufficient to assign the penicillin production phenotype to a particular habitat, an assignment further complicated if we consider other penicillin producers of the genus *Aspergillus*, and even more so if other β-lactam antibiotic producers (*A. chrysogenum*, *Streptomyces* spp.) are taken into account.

In the work of Nielsen et al. [232], the patulin and yanuthone D BGCs are compared between the 24 *Penicillium* species analyzed. *P. chrysogenum* Wis. 54-1255 contains an incomplete patulin cluster, a feature shared with the phylogenetically close *P. flavigenum* and *P. coprophilum*. The cluster lacks the genes encoding the isoepoxidon dehydrogenase, which catalyzes a middle step in the patulin biosynthetic pathway, and hence *P. chrysogenum* does not produce this mycotoxin. However, the presence in this cluster of the PatK gene, encoding a polyketide synthase, allows the biosynthesis of 6-methylsalicylic acid (6-MSA), which can be a precursor of other secondary metabolites, such as the antibiotic yanuthone D. A complete cluster for yanuthone D biosynthesis is present in the *P. chrysogenum genome*, and the synthesis of the compound was confirmed in this study [232].

Guzmán-Chávez et al. [235] made a graphic compilation of the BGCs present in the genome of *P. chrysogenum* Wis. 54-1255, locating the clusters at the relative positions they occupy in the chromosomes and assigning each cluster to the corresponding final product in the cases in which it is known. Up until 2018, only 14 of the total 50 clusters present in the genome had an assigned final product, and most of these, eight exactly, contained an NRPS encoding gene, among them the penicillin cluster.

Along with penicillin, 6-MSA, yanuthone D, and the pigments sorbicillin and chrysogenin (commented in previous sections), *P. chrysogenum* also produces mycotoxins such as roquefortine C, andrastin A, and PR-toxin. These mycotoxins are usually found in environments from which *P. chrysogenum* strains are isolated, e.g., paper from antique documents in historical archives [236]. Roquefortine C is the trademark metabolite of the blue cheese fungus *P. roqueforti*. *P chrysogenum* possesses a roquefortine C biosynthetic cluster consisting of seven genes, including the nonribosomal cyclodipeptide synthetase gene responsible for the biosynthesis of the first intermediate, Cyclo-His-Trp [237]. Roquefortine C would be synthesized in four enzymatic steps (three according to other authors, see below). In between the four genes encoding these enzymes are two other genes that extend the pathway and are responsible for the biosynthesis of the structurally related compound meleagrin [237]. Alternative branched roquefortine C/meleagrin pathways have been proposed by Ali et al. [238] and Ries et al. [239]. In various strains of *P. roqueforti*, a truncated version of the *P. chrysogenum* roquefortine C cluster is present that lacks a central region containing two genes indispensable for meleagrin biosynthesis [240]. The authors concluded that the original cluster, still present in *P. chrysogenum*, was trimmed down to a shorter cluster in *P. roqueforti*, leading to the synthesis of roquefortine C as the final product instead of meleagrin.

Regarding andrastin A, a compound showing anti-tumoral activity, Matsuda et al. [241] identified its biosynthetic cluster consisting of 11 genes and spanning a region of 30 kb in the genome of *P. chrysogenum*. The authors managed to reconstitute the pathway in a heterologous host, *A. oryzae*, by transferring a total of nine genes, including the backbone forming PKS gene *adrD*, resulting in the synthesis of andrastin A. A nearly identical andrastin A cluster is present in *P. roqueforti*, which totally conserves synteny with that of *P. chrysogenum* but for the absence of one gene of unknown function [242].

The presence of the PR-toxin in stored grains and blue cheeses is a potential problem due to the production of this toxin by *P. roqueforti*. A DNA region containing four genes involved in PR-toxin biosynthesis was first cloned in *P. roqueforti* [243]. Four highly similar orthologous genes were then identified in the genome of *P. chrysogenum*, which were part of a BGC comprising a total of 11 genes [243]. These genes remained nearly silent in typical cultures for penicillin production, but PR-toxin production could be induced in cultures on hydrated rice medium. Interestingly, in strain Wis. 54-1255 npe10, which lacks the 56.9 kb region containing the penicillin cluster, a 2.6-fold increase in PR-production was observed with respect to strain Wis. 54-1255, while the production was restored to the original levels in a Wis. 54-1255 npe10 derivative in which the three penicillin biosynthetic genes had been reintroduced [243]. Therefore, it seems that penicillin and PR-toxin production are inversely correlated.

The conidiation inducer conidiogenone, first identified in *Penicillium cyclopium* [244,245], is also produced by *P. chrysogenum* [246]. The authors identified one gene presumably involved in the biosynthesis of conidiogenone, gene Pc20g10860, in a Blast search using the bifunctional terpene synthase PaFS from *Phomopsis amygdali* as query, on the assumption that conidiogenone and phomopsene share a common biosynthetic pathway. A second gene, Pc20g10870, encoding a cytochrome P450 oxidase, was found adjoining the first one in opposite orientation. Both genes were introduced in *A. oryzae*, and a compound was identified whose NMR data and optical rotation value were in good agreement with those of conidiogenone. The conidiogenone BGC is predicted to consist of six to seven genes, and BGCs containing orthologs of Pc20g10860 and Pc20g10870 have been identified in the genomes of ten *Penicillium* species [246].

For the remaining secondary metabolites produced by *P. chrysogenum* Wis. 54-1255 whose biosynthetic cluster has been identified see Guzmán-Chávez et al. [235,247] and references therein. The number of 50 BGC in the genome of strain Wis. 54-1255 is, nevertheless, an underestimation. BGCs not containing typical genes of the kind of NRPS, PKS, or DMATs may be overlooked by secondary metabolite BGC search tools such as SMURF or antiSMASH. An example of this is the identification in the genome of strain Wis. 54-1255 of a putative BGC containing six genes orthologous to some genes present in the ustiloxin B and kojic acid BGCs of *A. flavus* and *A. oryzae*, respectively, which indicates that Wis. 54-1255 could synthesize a compound chemically related to them [248].

### 9.2. Genome Mining and Its Application in P. chrysogenum

BGCs usually contain one or more genes encoding scaffold-synthesizing enzymes such as PKS or NRPS, responsible for the formation of the structural backbone of the metabolite. Algorithms designed to search for BGCs in fungal genomes locate these identifying genes and analyze the ontology of the adjoining genes, thus predicting the presence of a BGC. Although biosynthetic genes being grouped in clusters greatly facilitates the assignment of genes to a BGC, in silico analysis alone cannot predict the precise extent of the cluster, neither can it provide information on whether the product is actually being synthesized [249]. There exist many cryptic BGCs that remain silent under standard laboratory conditions [231,250,251]. Once BGCs have been identified in a genome, the next challenges are to assign known secondary metabolites to them and to activate transcription of those that remain silent [98,251]. Activation of silent clusters can be endogenous or exogenous (heterologous hosts). Endogenous activation is limited to fungal species with a well-established system of manipulation and study of their genetics [231]. In these cases, various activation strategies have been successfully applied [250,252]. For those fungi in which molecular genetics tools are not sufficiently developed, an alternative method is to place and express their BGCs in heterologous systems [99,100,253]. Other genome-mining approaches follow a “chemistry first” strategy, targeting specific chemical features or biological properties of bioactive molecules and then searching for genes containing domains expected to participate in the biosynthesis of such chemical structures, and from here, locate putative BGCs in the genome that could be responsible for their synthesis [254].

In the case of *P. chrysogenum* Wis. 54-1255, there are 36 BGCs whose product has not yet been identified according to Guzmán-Chávez et al. [235]. So far, the use of strategies for awakening silent endogenous BGCs in *P. chrysogenum* have been limited to epigenetic manipulation to alter the transcriptional accessibility to the BGCs. HdaA, a class 2 histone deacetylase, is involved in the regulation of BGCs that are located near the telomeres in *A. nidulans*, such as the penicillin and sterigmatocystin clusters [255]. The *P. chrysogenum hdaA* gene, orthologous to *A. nidulans hdaA*, has recently been characterized as a key regulator of secondary metabolism [247]. Δ*hdaA* mutants showed significant changes of secondary metabolite gene expression including PKS and NRPS genes with known and unknown function. The absence of HdaA induced the activation of the sorbicillinoids BGC, activating transcription mainly of the *sorA*, *sorB,* and *sorC* genes. In contrast, the *hdaA* deletion caused a decrease in chrysogine levels, as a consequence of the reduction in transcription of the *chyA* gene. This effect on sorbicillinoids was also described in an endophytic strain of *P. chrysogenum* [256]; the deletion of *hdaA* in this strain also produced an increase in the meleagrin/roquefortine production, thus having opposite effects on the expression of different BGCs. Interestingly, HdaA mediates the transcriptional crosstalk among sorbicillinoids biosynthesis and other BGCs, since a new compound was detected only under conditions of sorbicillinoids production [247]. These results constitute the first report of regulatory crosstalk between BGCs in *P. chrysogenum*.

Another approach to induce expression of BGCs is the use of inhibitors of proteins participating in epigenetic regulation. Zhen et al. [257] used the histone-deacetylase inhibitor valproate sodium to elicit the production of new secondary metabolites from *Penicillium chrysogenum* HLS111, a strain isolated from the sponge *Gelliodes carnosa*, collected from Lingshui Bay, Hainan Province, China. Three new heterodimeric tetrahydroxanthone-chromanone lactones were identified, named chrysoxanthones A–C, which exhibited moderate antibacterial activities against *Bacillus subtilis*.

### 9.3. Secondary Metabolites Isolated from Other P. chrysogenum Strains

Many reports in the literature describe the production of different secondary metabolites, often with assigned bioactivities, by fungi identified as *P. chrysogenum*. These *P. chrysogenum* strains have been isolated from many different habitats, including endophytic and extreme environments. On very few occasions, these metabolites have been assigned a particular BGC or a gene involved in the biosynthesis since most of the works are focused on the detection and identification of the metabolites by analytical methods. Some of these metabolites may correspond to the “orphan” BGCs identified in the strain Wis. 54-1255 genome [235], but on other occasions, they may be metabolites produced by different BGCs that are present specifically in the isolated strains and absent in Wis. 54-1255. In Table 1, we make a compilation of secondary metabolites isolated from different *P. chrysogenum* strains, including information on their biological activities.

## 10. A Growing Toolkit

From the initial isolation/assay empirical techniques to the current molecular and synthetic biology tools, a number of methodologies have been applied to *P. chrysogenum* for strain improvement and to study penicillin biosynthesis and its regulation. In this section, we will summarize the most relevant.

### 10.1. Recombination Techniques

The apparent lack of a sexual cycle in the *P. chrysogenum* strains first used for industrial production of penicillin (later reclassified as *P. rubens*) precluded the possibility of using sexual recombination of desirable traits as a way to improve the penicillin production process. Thus, CSI programs based on random mutation and mutant selection became the prevalent strategy to effectively improve production yields for decades. However, attempts to recombine interesting characteristics from different strains were also carried out.

A parasexual cycle was described in *P. chrysogenum* [285] after its discovery in *A. nidulans* and *A. niger* [286]. The recombination frequency of this parasexual cycle is, however, much lower than that observed in the *A. nidulans* sexual cycle. The parasexual cycle was applied for strain improvement, and some *P. chrysogenum* heterozygous diploid strains could be isolated with enhanced penicillin production capacity [287,288,289].

Another strategy to recombine desirable characteristics of different strains is protoplast fusion [290] (reviewed by Gnanam [291] and Hospet et al. [292]). In *P. chrysogenum*, the Panlabs strain improvement program included steps in which protoplast fusion was employed; the characteristics of an improved penicillin-producing strain that showed poor sporulation and poor seed growth were modified by protoplast fusion and backcrossing with a low-producing strain yielded a higher-producing strain with better sporulation and growth [17]. In another example, Chang et al. [293] utilized protoplast fusion to combine the desirable qualities of two strains differing in colony morphology and the ability to produce penicillin V (the desired product) and OH-V penicillin (an undesirable product); analysis of 100 stable colonies showed that two possessed the desirable morphology, high penicillin V and low OH-V penicillin productivities. Interspecific protoplast fusion has also been used in the β-lactam industry; by fusing protoplasts from strains of *P. chrysogenum* and *Cephalosporium acremonium*, Chen et al. [294] isolated a strain capable of producing a novel β-lactam antibiotic.

The discovery that industrial penicillin-producing strains belong to a mating-type of *P. rubens* and that a sexual life cycle can be induced between these strains and wild strains of the opposite mating-type, generating viable ascospores with recombinant characteristics (described in a previous section), opened the possibility to use sexual crossing as a tool for strain improvement and other applications [295].

### 10.2. Genetic Transformation

Development of an efficient transformation system was key to the study of the function of genes involved in penicillin biosynthesis and would have numerous applications in subsequent years, among them the implementation of strategies for strain improvement.

The first systems for *P. chrysogenum* transformation were based on the isolation of auxotrophic mutants and complementation of the metabolic deficiency with an integrative plasmid carrying the appropriate gene. The protocols consisted of the isolation of protoplasts with a commercial cocktail of cell wall-degrading enzymes and introduction of the plasmid using polyethilenglycol (PEG), followed by regeneration of transformants on selective medium containing an osmotic stabilizer. Cantoral et al. [296] set up a protocol for efficient transformation of *P. chrysogenum* using a *pyrG* mutant strain previously obtained by resistance to 5-fluoroorotic acid (5-FOA) [297]. The *pyrG* mutants are unable to grow on minimal media without added uridine since they lack orotidine 5′-monophosphate (OMP) decarboxylase. Complementation of this mutation was achieved with plasmids carrying the *pyr4* gene of *Neurospora crassa*, homologous to the *pyrG* gene, allowing the isolation of 1–4 × 10^3^ stable transformants per μg of DNA [296,297]. Later, the own *P. chrysogenum pyrG* gene [298] would be routinely used as selection marker. Another auxotrophy complementation strategy was devised by Sánchez et al. [299] based on the isolation of tryptophan auxotrophs after UV-radiation and the use of a plasmid containing the *P. chrysogenum trpC* gene. A similar strategy based on the *trpC* gene was independently developed by Picknett et al. [300], reporting an efficiency of 300–1800 transformants per μg DNA. The *niaD* gene has also been used as auxotrophy complementation marker, after obtaining spontaneous *niaD* mutants by chlorate resistance [301].

Dependency on the use of auxotrophic strains was overcome with the development of transformation systems based on the use of antibiotic resistance and nutrient utilization markers. Beri and Turner [302] used the *A. nidulans* acetamidase gene (*amdS*) to transform *P. chrysogenum* at low frequency, and the same nutrient utilization marker was used for co-transformation by Kolar et al. [303]. Bull et al. [304] used the *P. chrysogenum oliC13* gene, encoding an oligomycin-resistant ATP synthase subunit 9, as dominant selection marker for *P. chrysogenum* transformation based on oligomycin resistance. The authors obtained a significant number of transformants, but the strategy is hindered by the necessity to get multicopy transformants due to the presence of the endogenous oligomycin-sensitive allele. Transformation with the *Streptoalloteichus hindustanus* phleomycin resistance (*ble*) gene fused to a fungal promoter would become standard in *P. chrysogenum* once this strategy was first developed by Kolar et al. [303]. Additional transformation systems were developed based on G418 (Geneticin^®^), sulphonamide, benomyl, and Terbinafine resistance [305,306,307,308].

The presence of antibiotic resistance genes after transformation hinders the introduction of new constructs in the cell because a different resistance marker should be used in subsequent experiments, and the resistance genes might have unwanted effects. To overcome this, Kopke et al. [309] developed a recombination strategy for marker recycling based on the *S. cerevisiae* FLP/FRT system, thus avoiding the presence of heterologous genes in transformants and in knockout strains.

Isolation of protoplasts and PEG-mediated transformation go on being the preferred method for introduction of exogenous DNA in *P. chrysogenum*, but a few other transformation methodologies have also been successfully adapted to the fungus. Electroporation of protoplasts was used by García-Rico et al. [207] to transform a strain resistant to conventional PEG-mediated transformation. Sun et al. [310] reported the use of *Agrobacterium tumefaciens*-mediated transformation (ATMT). de Boer et al. [311] used also an ATMT approach to investigate gene disruption efficiency (see below). Recently, Vu et al. [312] developed an improved ATMT system for *P. chrysogenum* with two different selection markers, which yielded 5009 ± 96 transformants per 10^6^ spores.

### 10.3. Autonomously Replicating Vectors

Exogenous DNA integrates in the *P. chrysogenum* genome when conventional vectors are used for transformation. However, the use of vectors with the capacity for autonomous replication is of great interest for some purposes and has found important recent applications.

The first vectors for *P. chrysogenum* claimed to have autonomous replication capacity were based on mitochondrial DNA sequences [305]; however, this strategy was inefficient and did not have further development. The finding of a genomic DNA sequence in *A. nidulans* conferring autonomous replication capacity, named AMA1 (Autonomously Maintained in *Aspergillus* 1), entailed an important breakthrough for the development of functional fungal vectors with this property [313]. The AMA1 sequence was isolated from a genomic library based on its capacity to enhance 2000-fold the transformation efficiency over conventional integrative plasmids. AMA1-containing plasmids gave rise to sectorial colonies, suggestive of plasmid instability and loss. Structural analysis of AMA1 revealed that it was a 6.1 kb fragment formed by inverted repeats separated by a 0.6 kb central fragment and that it was the central part of a larger genomic structure formed by two inverted MATE (Mobile *Aspergillus* Transformation Enhancers) elements, which showed some features of mobile elements [314]. A HindIII 5.0 kb AMA1 fragment comprising the central region of the original 6.1 kb fragment was used to construct plasmids pAMPF2 and pAMPF21, carrying as selection markers the *pyrG* gene and the *ble* gene, respectively, to be used in *P. chrysogenum* [315]. The plasmids showed transformation efficiencies of 218,000 (pAMPF2) and 200 (pAMPF21) transformants per μg DNA, and the colonies of a proportion of transformants showed a wrinkled and sectorial phenotype. The mitotic stability was around 48%. Plasmids appeared as multimers (dimers and trimers) in the mycelium, with a copy number of around 35 per genome, and were easily recovered in *E. coli*. The recovered plasmids showed no rearrangements with respect to the original ones [315]. Both plasmids showed a very similar behaviour in the cheese and ham-growing fungus *P. nalgiovense*, with slightly higher mitotic stability [316].

AMA1-containing plasmids have been used for different purposes in *P. chrysogenum*. Lamas-Maceiras et al. [116] used plasmid pAMPF9L, containing one of the two inverted repeats of 2.2 kb [315], to overexpress the *phl* gene, on the basis of the high number of plasmid copies in the cell. More recently, plasmid pAMPF21 has been used to construct a series of vectors to implement different CRISPR/Cas9 strategies for genome editing in *P. chrysogenum* [317] and to obtain vectors for modular assembly of functional genetic elements in a synthetic biology approach to develop *P. chrysogenum* into a cell factory [318]. These strategies will be discussed below, in the sections dealing with synthetic biology.

### 10.4. Tools for Gene Overexpression

Obtaining transformants with multiple copies of a gene is a valid strategy for gene overexpression in *P. chrysogenum*. The vector can be integrative [319,320,321] or autoreplicative [116]. An improved strategy is overexpression using strong promoters. García-Estrada et el. [119] used the *A. awamori gdh* gene promoter to overexpress the *ppt* gene, encoding phosphopantetheinyl transferase (see below). The endogenous *P. chrysogenum gdh* gene (*gdhA*) was cloned and its promoter fused to the three penicillin biosynthetic genes to analyze the expected enhancement in enzyme activities and antibiotic production [322]. IPNS activity showed increases of 10–30% in monocopy transformants overexpressing the *pcbC* gene, whereas increases of 10–50% in AAT activity were obtained with overexpression of the *penDE* gene. Penicillin production was increased by an average of 20% in these transformants with respect to the parental strain Wis. 54-1255. The *pcbC* gene promoter is known to possess high transcription efficiency and has been used for gene overexpression in some instances. Kiel et al. [132] placed the gene encoding Pc-Pex11p, a dominant peroxisome membrane protein, under control of the *pcbC* promoter, which led to massive proliferation of tubular-shaped microbodies and a 2- to 2.5-fold increase in penicillin production.

Polli et al. [323] assayed the performance of a series of endogenous and exogenous promoters using the eGFP-encoding gene as reporter, obtaining a ranking of promoter strengths with the promoter of the Pc20g15140 gene, encoding a secreted serine protease, at the top of the list. The most complete list of promoters that can be used for overexpression in *P. chrysogenum* is provided by Mózsik et al. [318], a list that also includes elements such as UASs, core promoters, terminators, and other tools for synthetic biology. Regarding inducible promoters, the *P. chrysogenum xylP* gene promoter was tested for activity using the *uidA* reporter gene, showing induction by xylose (and xylan) and repression by glucose [324]. Although the authors used the *xylP* promoter for inducible silencing of the *nre* gene, its application for inducible gene overexpression should also be possible.

### 10.5. Utilization of Reporter Genes and Targeted Integration to Obtain Monocopy Transformants

Studies on the promoter activity of the penicillin genes based on reporter gene fusions have been of great importance to elucidate the signals and culture conditions that regulate the expression of the genes. This strategy proved also to be highly valuable to identify and characterize regulatory elements present in them. The *lacZ* gene fused to the *pcbAB* gene promoter has been used to study pH regulation [183], identify regulatory regions important for transcription [189], and analyze regulatory elements involved in carbon catabolite repression [178]. Gutiérrez et al. [325] used fusions of the three penicillin gene promoters to *lacZ* to study carbon source and pH regulation. The β-glucuronidase-encoding gene, *uidA*, has also been used as reporter to study carbon and nitrogen source regulation of the *pcbAB* and *pcbC* gene promoters [179].

The aforementioned studies required a single copy of the gene fusions integrated at a specific site for comparative purposes, avoiding the effect of the genetic context in promoter activity as well as preventing the possible random disruption of some endogenous gene. Feng et al. [179] developed a gene-targeting strategy based on the presence of a truncated *niaD* gene in the vector carrying the gene fusion and a host strain with a mutant *niaD* gene. Viable prototrophic transformants occur only if homologous recombination at the mutant *niaD* gene locus takes place, giving rise to a complete and functional *niaD* gene. The presence of a single copy of the gene fusion is then confirmed by Southern blot. The same strategy was used by Gutiérrez et al. [325] with a vector carrying a mutated *pyrG* gene and a uridine auxotrophic host strain with a mutant *pyrG* gene.

### 10.6. Targeted Integration to Obtain Gene Knockout Strains

In *P. chrysogenum*, plasmid integration by homologous recombination occurs at a very low frequency in comparison to ectopic integration by other recombination mechanisms. This makes gene disruption and deletion strategies inefficient and time-consuming, requiring large DNA fragments to target the specific locus [207,326]. In order to improve gene disruption efficiency, different strategies have been implemented. Ectopic integration in fungi is presumed to be the result of recombination processes such as non-homologous end joining (NHEJ). To decrease the frequency of these events, the genes *hdfA* and *hdfB*, homologous to human *ku70* and *ku80* and involved in NHEJ, have been inactivated in some *P. chrysogenum* strains. The genes were deleted by conventional strategies, and the resulting mutant strains showed a dramatic increase in the frequency of gene disruption events by homologous recombination, from < 1% to 35–65% [327,328]. Snoek et al. [327] obtained Δ*hdfA* and Δ*hdfB* mutants from a high-penicillin-producing strain, DS17690. The authors reported a reduced fitness when the Δ*hdfA* strain was compared to the reference strain; specifically, it showed a lower growth rate, although biomass specific penicillin G production was not affected. Hoff et al. [328] obtained a Δ*hdfA* mutant from another high-producing strain, P2niaD18, reporting that the mutation did not cause significant changes in penicillin production, growth rate, or sensitivity towards mutagens, but it caused an induced expression of genes involved in stress responses. Another gene involved in NHEJ in fungi is *lig4*, encoding a protein that forms part of a heterotrimeric DNA ligase 4 complex that seals the gap produced in a double strand break after damage recognition by the dimer Ku70-Ku80. de Boer et al. [329] obtained a Δ*lig4* mutant from strain DS17690 and compared its gene-targeting efficiency with the Δ*hdfA* strain previously obtained by Snoek et al. [327], observing a higher efficiency in the disruption of one of the three genes tested.

For gene deletion in *P. chrysogenum*, a double marker approach is frequently used [207]. Two different marker genes are employed; one substitutes the gene or part of the gene to be deleted, and the other is placed outside the deletion cassette in an arrangement: marker_1::5′-homologous_region::marker_2::3′-homologous_region. Selection is then performed for conservation of marker 2 and loss of marker 1, expecting in this way an increase in the proportion of transformants where homologous recombination took place. Another strategy used for gene deletion in *P. chrysogenum* has been the bi-partite approach or split marker. In this strategy, two different linearized plasmids are employed, each containing overlapping fragments of the marker gene at the edge; in vivo reconstitution of the marker will preferentially occur when the 5′- and 3′-homologous regions placed beside the marker gene fragment in each plasmid recombine with the endogenous gene intended to be deleted. de Boer et al. [329] used a split marker approach first to obtain a Δ*lig4* strain (see above) and then to inactivate (deleting a central fragment) the *niaD* gene. The authors reported increases in deletion efficiency of 2- to 2.5-fold when using the split marker approach with respect to a conventional gene deletion strategy with one plasmid. The combination of the Δ*lig4* genetic background and the split marker strategy raised gene deletion frequency up to 90%. The split marker approach has also been used in combination with ATMT, transferring the two plasmids, each carrying one of the two marker gene overlapping fragments, via the same *Agrobacterium* host [311]. This strategy yielded a gene targeting efficiency of 97%, without additional ectopic T-DNA insertions in most cases, and was proven to work with conidia, thus avoiding the need for protoplast isolation.

The development of CRISPR-based gene inactivation strategies for *P. chrysogenum* [317] has entailed an important step forward for the isolation of knockout mutants. This strategy will be discussed in next sections, in the context of synthetic biology approaches.

### 10.7. RNAi-Mediated Gene Silencing

Due to the difficulties for gene knockout in *P. chrysogenum*, an alternative strategy to quickly analyze the function of a gene is RNAi-mediated gene silencing. This strategy is also convenient when the effect of knocking out a gene is too drastic and produces an extreme phenotype (severely hampered growth for instance) that may mask the function of the gene in a specific process. RNAi-mediated gene silencing in *P. chrysogenum* was first applied by Ullán et al. [330] to knock down expression of the *pcbC* gene, resulting in a 70–82% decrease in the *pcbC* mRNA levels throughout the cultivation time with a concomitant reduction of penicillin G production of 70–85%. The strategy was based in the expected generation of a dsRNA after transcription from two opposite-oriented promoters of a DNA fragment inserted between them. The best results have been obtained with DNA fragments 400–500 bp in length (our own results). This strategy has been successfully applied to study the function of genes encoding transcriptional regulators and other proteins involved in penicillin biosynthesis and other processes [120,135,178,193,218].

Janus et al. [331] found evidence of the existence of RNAi-mediated gene silencing in *P. chrysogenum*. The authors identified two putative Dicer-encoding genes in the genome, *Pcdcl1* and *Pcdcl2*, and developed a silencing strategy based on the generation of a transcript containing inverted repeats of a fragment of the target gene from a single promoter, which would form a dsRNA with a hairpin. Knocking down of the conidiation regulator *brlA* gene was demonstrated by Northern blot, with a concomitant reduction in conidia formation. When the same experiment was performed in a Δ*Pcdcl2* strain, no reduction in conidiation was observed. Further evidence for the existence of RNAi-mediated silencing was the detection of small RNAs, around 20–25 nucleotides long, which hybridized to a *brlA* probe [331]; these RNAs can be assimilated to siRNAs. A study of naturally occurring microRNA-like RNAs (milRNA) in *P. chrysogenum* using small RNA deep sequencing was performed by Dahlmann and Kück [332]. The authors reported the identification of 34 milRNAs, most of them with evidence of Dicer processing, and their absence in a double Δ*dcl2*Δ*dcl1* strain.

## 11. Improving on the Improved: Engineering *P. chrysogenum* to Increase Penicillin Production and Synthesize Cephalosporin Precursors

In previous sections, we described how CSI programs had managed to increase penicillin production levels by more than three orders of magnitude and discussed some of the genetic changes related to penicillin production that are found in high-producing industrial strains. The arrival of the genetic engineering era with the development of an array of tools for genetic manipulation (see previous section) and the increasing knowledge on the metabolic and cellular aspects of penicillin biosynthesis allowed researchers to design more directed strategies for improving penicillin production levels, incorporating approaches of metabolic engineering. We will discuss some of these strategies in this section.

### 11.1. Increase of the Copy Number of the Penicillin Biosynthetic Genes

Early investigations performed with *P. chrysogenum* strains obtained from CSI programs and producing high levels of penicillin showed that the increase in gene dosage of the penicillin genes underlies the high yields of penicillin [52,53,54]. Therefore, the re-introduction and overexpression of genes from the penicillin gene cluster appeared, in principle, as a promising way to increase penicillin production. The first attempt in this direction was unsuccessful, Skatrud et al. [333] introduced extra copies of the *A. nidulans pcbC* gene in an industrial strain of *P. chrysogenum* but no increase in penicillin production was observed. Later, Veenstra et al. [319] achieved better results by introducing extra copies of a DNA fragment containing the *P. chrysogenum pcbC* and *penDE* genes into the Wis. 54-1255 strain; the authors managed to increase the production level of penicillin V by up to 40%.

Before the availability of OMICs methodologies, bottlenecks for penicillin production were detected mainly by carbon flux analysis [61,334,335]. Regarding the three biosynthetic steps of the penicillin pathway, the first two steps were proposed as limiting for high levels of production [336,337]. Based on these results, Theilgaard et al. [320] analyzed the effect of introducing extra copies of the penicillin biosynthetic genes in different combinations in the strain Wis. 54-1255. The authors obtained a 44–82% increase in penicillin production in transformants containing one or two additional copies of the *pcbC* or *penDE* genes, and the best results were obtained when extra copies of the three biosynthetic genes were introduced (176% increase). The authors also concluded that an increase in penicillin productivity is better achieved when a balanced amplification of both the ACVS and IPNS activities occurs. This strategy is, nevertheless, unlikely to be effective for industrial production. As previously mentioned, industrial strains already contain extra copies of the penicillin gene cluster, and while there is a correlation between number of copies and production levels at low copy number, this correlation is lost above a threshold of around five copies [57,58]. However, Nijland et al. [58] identified the IAT as a possible bottleneck in strains containing several copies of the penicillin cluster. The protein levels of this enzyme did not follow a linear correlation with the number of copies of the gene and became saturated at low copy number, which did not occur with the other two enzymes of the pathway. As a result, an accumulation of IPN was observed in the broths. Based on these results, Weber et al. [136] designed a strategy to increase the levels of IAT by introducing additional copies of the *penDE* gene in strains with different numbers of copies of the penicillin cluster. When up to 10 extra copies of the *penDE* gene were introduced, penicillin production increased, even in strains containing multiple copies of the penicillin cluster; however, introduction of a higher number of copies had the opposite effect and the penicillin levels dropped, concomitant with a rise of 6-APA in the broths. These results indicate that the number of copies of the penicillin biosynthetic genes must be balanced and that fine-tuned expression of the *penDE* gene is important to achieve an increase in penicillin production. The IAT activity is indeed limiting in strains with a high copy number of the penicillin cluster, and its overexpression improves penicillin production, but above a certain level of activity, the effect turns negative, probably because the hydrolase activity of the enzyme becomes prevalent and removes the PAA lateral chain from penicillin and/or IPN.

### 11.2. Increase of Precursor Availability

Enzymes involved in penicillin biosynthesis require precursors that are produced by other metabolic pathways. Because the metabolic fluxes of these pathways determine the availability of precursors for the biosynthesis of penicillin, the appropriate channelization of key metabolites from these pathways could improve the production of penicillin. Therefore, one of the main metabolic engineering strategies to improve penicillin production has been increasing the availability of precursors of the penicillin pathway, such as the three amino acids forming the tripeptide ACV or the PAA that is incorporated in the last step of penicillin G biosynthesis.

α-aminoadipic acid (α-AAA) incorporates into the tripeptide ACV and is one of the main precursors for penicillin biosynthesis. However, α-AAA also participates in the biosynthesis of lysine, it is the branching intermediate where the pathways for the biosynthesis of penicillin and lysine diverge. Therefore, in *P. chrysogenum*, the α-AAA pool must feed both pathways, and blocking the branch leading to lysine would channelize the carbon flux to penicillin. Casqueiro et al. [338] carried out the disruption of the *lys2* gene, encoding aminoadipate reductase, the first enzyme in the pathway for the conversion of α-AAA to lysine. The disrupted mutants were lysine auxotrophs, and showed up to a 3-fold increase in specific production (micrograms per milligram of biomass) of penicillin with respect to the parental strain.

Another example of flux channelization was the increase in the availability of phenylacetyl-CoA, which is a precursor required in the last step of the penicillin biosynthetic pathway, that is, the replacement of the α-aminoadipyl side chain by a phenylacetate residue [81]. A key gene in the activation of PAA to phenylacetyl-CoA is *phl*, which encodes an aryl-capping enzyme [116]. The increase in the copy number of *phl* by transforming the fungus with an autonomous replicating plasmid carrying the gene resulted in an 8-fold increase in phenylacetyl-CoA ligase activity and a 35% increase in penicillin production.

The phosphopantetheinyl transferase does not participate in the biosynthesis of precursors but it is essential for penicillin production since it is responsible for the incorporation of the phosphopantetheine coenzyme to the ACVS, which is inactive without this amino acid carrier that transfer intermediates between the enzyme modules. The *ppt* gene of *P. chrysogenum* was cloned and shown to encode a phosphopantetheinyl transferase required for penicillin and lysine biosynthesis but not for fatty acid biosynthesis [119]. Overexpression of the *ppt* gene by fusion to the *gdh* promoter resulted in a 3-fold increase of transcript levels and a 30% increase in penicillin production with respect to the parental strain Wis. 54-1255.

### 11.3. Improvement of Precursor and Penicillin Transport

Penicillin biosynthesis is a process that occurs in different cellular compartments [126], hence facilitating the transport of intermediaries across internal membranes could lead to an increase in penicillin production. The last step in penicillin G biosynthesis is performed in the peroxisomes, and requires the import of IPN and PAA from the cytoplasm to this organelle, which is carried out by the MFS transporters PenM and PaaT, respectively [134,135]. The overexpression of the *penM* gene in strain Wis. 54-1255 yielded strains with increased levels of penicillin production, ranging from 169% to 236%, as compared to the control strain [134]. Similarly, strains overexpressing the *paaT* gene showed a 40–100% increase in the levels of produced penicillin in strain Wis. 54-1255 [135] and similar increases in the wild strain CGMCC 3.5129 [159].

Regarding the increase of transporters for the secretion of penicillin to the extracellular medium, the obtained results are not conclusive. Ullán et al. [339] expressed the *cefT* gene from *A. chrysogenum* in three strains of *P. chrysogenum*, including one previously engineered for the production of cephalosporins, named strain TA98 [340] (see below). The *cefT* gene encodes a transmembrane MFS protein which was shown to be involved in CPC secretion [158] as well as secretion of other cephem intermediates of the CPC biosynthetic pathway [153]. As a result, Ullán et al. [339] found that, although the expression of *cefT* in *P. chrysogenum* increased the secretion of hydrophilic penicillins (IPN and penicillin N), the production of benzylpenicillin was drastically reduced. It should be mentioned that the real mechanism for the secretion of penicillin in *P. chrysogenum* is currently unknown and, indeed, it is a matter of controversy (discussed in a previous section). Apparently, this mechanism requires autophagy of peroxisomes and vesicles traffic, but it would not require the participation of any transporter protein [126].

Since penicillin biosynthesis occurs in different intracellular locations, the increase of the capacity of relevant compartments might increase its production. Kiel et al. [132] performed a demonstration of this principle by overexpressing the peroxin protein Pc-Pex11p, resulting in a massive proliferation of tubular-shaped microbodies and an increase in the penicillin levels in the culture broth of about 2-2.5-fold as compared with the parental strain. At this point, it would seem feasible that another strategy to increase penicillin production could be the re-location of the penicillin biosynthetic enzymes directly into more suitable, in principle, intracellular compartments. However, and as has been recently pointed out by Martín [126], attempts aiming for the modification of the spatial organization of penicillin enzymes have been unsuccessful. There seem to be inherent difficulties in moving enzymes from a compartment to another which have not been resolved yet.

### 11.4. Concluding Remarks on Strain Improvement Using Genetic Engineering

The mentioned strategies worked well when applied to strains with low capacity of penicillin production and one copy of the penicillin biosynthetic cluster. However, industrial strains are more complex and often the strategies fail when trying to be applied to them. The channelization process performed by genetic engineering may have already been achieved through random mutation during the CSI programs, and the presence of many copies of the penicillin genes precludes, or at least hinders, the success of strategies based on introducing extra copies, although balanced overexpression of the *penDE* gene resulted successful in strains with several copies of the cluster [136].

Past carbon flux analyses and recent OMICs studies are coincidental in pointing to primary metabolism and the supply of NADPH, cysteine and ATP as key factors for high levels of penicillin production ([25,38] and references therein). Therefore, future metabolic engineering efforts could target the pathways that would increase the availability of these metabolites. Finally, although a good deal of regulatory pathways and transcription factors are now known to regulate transcription of the penicillin genes and penicillin production (see above), no efforts, to our knowledge, have been made to improve productivity by manipulating these regulatory mechanisms, at least that have been made public, even though at laboratory scale, modification of some of them have shown promising results [178,209].

### 11.5. Engineering P. chrysogenum Pathways for the Production of Cephalosporins

Taking into account the improved ability for the production of β-lactam antibiotics of industrial *P. chrysogenum* strains, it appeared as natural the re-engineering of these strains for the production of cephalosporins or precursors of semisynthetic cephalosporins. One of the first approaches was the production of CPC by re-engineering of the *P. chrysogenum* strain npe6 [340]. This strain does not produce benzylpenicillin and accumulates IPN due to the lack of IAT activity [341,342]. Using this strain as recipient, Ullán et al. [340] introduced and expressed four genes from *A. chrysogenum* (*cefD1*, *cefD2 cefEF* and *cefG*), which encode the enzymes required for the successive transformation of IPN into IPN-CoA, penicillin N, deacetylcephalosporin C (DAC), and CPC, respectively. The obtained strain was named TA98 and produced and secreted IPN, penicillin N, and DAC to the cultured broth, but no CPC, which was produced but intracellularly retained.

The previous result highlights the importance of extracellular secretion in cephalosporin production. In this regard, two works addressed the possibility of expressing the *A. chrysogenum cefT* gene in *P. chrysogenum* strains engineered for the production of cephalosporins to transport cephalosporin or its precursors out of the cell. Ullán et al. [339] expressed the *cefT* gene in the *P. chrysogenum* TA98 strain and observed an increase in the secretion of DAC to the extracellular medium, but no CPC was detected in significant amounts. A rapid deacetylation of CPC to DAC in the culture broths may occur; this reaction actually takes place in *A. chrysogenum* and one enzyme with acetylhydrolase activity has been characterized which is involved in this process [343]. Mis-targeting of the DAC-acetyltransferase enzyme in *P. chrysogenum* cannot be excluded either. Additionally, the broad range of substrate specificity of CefT as hydrophilic β-lactam transporter must be taken into account; along with CPC, CefT has been shown to export penicillin N, IPN, DAC and DAOC [153], and, possibly as a consequence of this, overexpression of *cefT* in high CPC producing strains resulted in decreased amounts of CPC in the broths [153,344]. On the other hand, Nijland et al. [345] followed the same approach with an industrial strain of *P. chrysogenum* engineered for the production of adipoyl-7-amino-3-carbamoyloxymethyl-3-cephem-4carboxylic acid (ad-7-ACCCA), a precursor of semisynthetic cephalosporins (see below). As a result, they found that the heterologous expression of *cefT* resulted in an almost 2-fold increase in the levels of secreted ad7-ACCCA as compared with the parental strain, which supports the role of CefT as transporter of a broad range of β-lactam ring-containing compounds.

In relation to the production of precursors of semisynthetic cephalosporins, several efforts have been made for their production in engineered strains of *P. chrysogenum*. In all these cases, the metabolite adipoyl-6-aminopenicillanic acid (ad-6-APA) plays a key role. ad-6-APA can be produced by *P. chrysogenum* when the fungus is fed with adipic acid. In the cell, adipic acid is activated to adipoyl-CoA and subsequently attached to IPN by the IAT, thus forming ad-6-APA, which can now enter the cephalosporin pathway [346]. From this starting point, Crawford et al. [347] obtained several *P. chrysogenum* strains engineered to produce precursors of semisynthetic cephalosporins when they were fed with adipic acid. In one of these strains, the authors introduced the *cefE* gene from *Streptomyces clavuligerus*, which encodes a DAC expandase that transforms ad-6-APA into adipoyl-7-aminodeacetoxy-cephalosporanic acid (ad-7-ADCA). The ad-7-ADCA produced by the *cefE*-containing *P. chrysogenum* strain can later be converted into 7-ADCA, a precursor of semisynthetic cephalosporins, by an in vitro enzymatic cleavage of the adipoyl side chain [347,348]. In another *P. chrysogenum* strain, Crawford et al. [347] introduced and expressed the *cefEF* gene from *A. chrysogenum*, encoding DAC expandase/hydroxylase, and obtained a strain producing ad-7-ADCA as well as adipoyl-7-aminodeacetylcephalosporanic acid (ad-7-ADAC). As happens with ad-7-ADCA, ad-7-ADAC can also be converted into the precursor of semisynthetic cephalosporins 7-ADAC by cleaving the adipoyl side chain with a suitable enzyme [347]. Finally, by introducing the *A. chrysogenum cefG* gene, encoding a DAC acyltransferase, the authors obtained a *P. chrysogenum* strain that can transform ad-7-ADAC into adipoyl-7-aminocephalosporanic acid (Ad-7-ACA), which, after the enzymatic elimination of the adipoyl side chain, can be converted into the precursor of semisynthetic cephalosporins 7-aminocephalosporanic acid (7-ACA).

It is known that a major problem associated with cephalosporins is their chemical instability [4]. With this in mind, Harris et al. [21] proposed that obtaining precursors carbamylated in the 3-position of the dihydrothiazine ring could offer important advantages for the production of semisynthetic cephalosporins. For this purpose, they expressed in *P. chrysogenum* the *cefEF* gene from *A. chrysogenum* along with *cmcH*, a gene encoding a carbamoyl transferase from *S. clavuligerus*. As a result, the authors achieved the production of adipoyl-7-amino-3-carbamoyloxymethyl-3-cephem-4carboxylic acid (ad-7-ACCCA), which could be used for the synthesis of semi-synthetic cephalosporins.

## 12. Great Expectations: *P. chrysogenum* Goes Synthetic

*P. chrysogenum* has withstood the test of time and remains one of the top microbial species in the biotechnological industry, but its history seems far from coming to an end any time soon. New developments are in sight, and some groups are doing excellent work on the path to turn *P. chrysogenum* into a platform for heterologous production of chemicals, stocked with its industrial capabilities acquired through decades of strain and bioengineering developments.

The idea of developing *P. chrysogenum* strains as platforms for heterologous production of metabolites is framed in the context of its previous history as a very successful industrial microorganism, highly improved for penicillin production and with fine-tuned bioengineering processes adapted to streamline production. As mentioned in previous sections, *P. chrysogenum* has been submitted to intense CSI programs, which resulted in strains optimized for high-level production of β-lactam antibiotics [4]. As a collateral result of these CSI programs, strains currently used in the industry have lost a great deal of their ability to synthesize natural products other than β-lactams [235]. Therefore, high-penicillin-producing strains show an altered landscape of secondary metabolites because the very high titers of β-lactam antibiotics reflect the optimal carbon and nitrogen flow towards their production, to the detriment of the production of other secondary metabolites. Thus, industrial *P. chrysogenum* strains start off with an advantage, having lost at least part of the burden that secondary metabolites other than β-lactams represent, which means that eliminating the ability to synthesize β-lactams should lead to the successful application of synthetic biology strategies for production of other heterologous compounds.

In recent years, several synthetic biology tools for filamentous fungi have been developed [349], and some of these tools have been applied to *P. chrysogenum*. In the last part of this review, we summarize recent advances to engineer *P. chrysogenum* using synthetic biology approaches.

### 12.1. Reprogramming P. chrysogenum for the Production of Alternative Secondary Metabolites

Previously, we mentioned the synthesis of cephalosporins in engineered *P. chrysogenum* strains. These developments can be considered as the first examples of reprogramming *P. chrysogenum* to produce other compounds. Recently, synthetic biology strategies have been applied for the production of compounds other than β-lactams in *P. chrysogenum*. McLean et al. [350] achieved the production of the cholesterol-lowering drug pravastatin in a reprogrammed strain without β-lactam antibiotic background that had been previously obtained by deletion of the penicillin gene clusters from a high-producing strain [21]. In this β-lactam-negative strain, the nine genes of the compactin gene cluster from *P. citrinum* were introduced, all of them under control of their native promoter and terminator regions. In addition, to optimize production of the desired product (compactin) and avoid the production of undesirable deacylated compactin as a by-product, a putative esterase encoded by the Pc15g00720 gene was deleted. Finally, the last step necessary to produce pravastatin, the hydroxylation of compactin, was achieved by the introduction of an evolved cytochrome P450 (compactin hydrolase) from *Amycolatopsis orientalis*. This enzyme was evolved to maximize the production of pravastatin, avoiding the production of its undesirable stereoisomer 6-epi-pravastatin. As a result of this complete reprogramming, *P. chrysogenum* was able to produce more than 6 g/L of pravastatin at a pilot production scale, a very improved yield as compared with the 2–3 g/L of pravastatin obtained in other microbial systems [351,352].

Following a similar strategy, Pohl et al. [353] obtained a *P. chrysogenum* strain in which the biosynthetic gene clusters for the production of penicillin, roquefortine, chrysogenin, and fungisporin were inactivated by consecutive gene deletion procedures. As expected, the resulting strain, named 4xKO, showed negligible levels of these four compounds. Using this strain as a platform, the authors expressed the heterologous calbistrin gene cluster from *P. decumbens*, recently described by Grijseels et al. [169]. High yields of decumbenone A, B, and C in the culture supernatants were obtained. The deletion of the aforementioned secondary metabolite clusters was performed using the CRISPR/Cas9 technology [353], which will be detailed in the next section.

The mentioned studies show that *P. chrysogenum* can be rewired for the production of novel natural products not naturally produced by the fungus, obtaining yields that may make the process competitive in the industry. Therefore, as suggested by Pohl et al. [353], *P. chrysogenum* can be converted in a platform for production of heterologous metabolites by using synthetic biology approaches.

### 12.2. Development of CRIPSR/Cas9 Technology and the Future of Synthetic Biology in P. chrysogenum

The classic genetic engineering strategies described in a previous section allow the development of synthetic biology approaches in *P. chrysogenum* but has limitations. For the full exploitation of what synthetic biology can offer, the availability of much more efficient technologies is required, among them technologies aiming for the elimination or edition of genes. As mentioned before, gene knockout by homologous recombination has a very low success rate in *P. chrysogenum*, which makes it very difficult to address ambitious synthetic biology programs based on this methodology in a reasonable time. The CRISPR/Cas9 technique has been a game changer in genetic engineering, with implications for biotechnology that are still in their first steps of exploration. As in other organisms, CRISPR/Cas9 allows precise and relatively quick genetic editing in fungi [354]. The application of this technique in *P. chrysogenum* was described by Pohl et al. [317]. The authors developed a complete toolkit for the edition of the *P. chrysogenum* genome, which includes the possibility of delivering the Cas9 protein with in vitro synthesized sgRNA as a pre-assembled ribonucleoprotein complex directly during the transformation, or alternatively expressing them from an autonomously replicating AMA1-based plasmid within the fungal cells. Using these approaches, Pohl et al. [317] managed to successfully edit several secondary metabolism genes, with or without the use of a marker-free donor DNA.

The availability of the CRISPR/Cas9 technique is expected to boost the application of synthetic biology tools in *P. chrysogenum*, allowing the exploration of other ways to re-engineer it. For example, an important goal in synthetic biology is the precise control of gene expression [355]. In this respect, orthogonal control devices for gene regulation in *P. chrysogenum* have been developed [356]. The devices consist of an expression cassette named “donor”, which expresses a synthetic transcription factor that includes a DNA-binding domain from the transcription factor that regulates the quinic acid gene cluster of *N. crassa*. In addition, the devices have a second cassette named “recipient”, consisting of a synthetic promoter containing quinic acid upstream activating sequence (QUAS) elements, upstream of a core promoter (CP) from different fungal genes. This synthetic promoter is activated by the binding of the synthetic transcription factor expressed by the donor cassette, allowing fine-tuning of expression levels of a reporter gene by varying the promoter that controls the expression of the synthetic transcription factor, the CPs in the synthetic promoter, and/or the number of QUAS elements. Thanks to the availability of the CRISPR/Cas9 technique, the control device was applied to the production of penicillin V. By replacing promoters of genes from the penicillin gene cluster with different versions of the synthetic promoter, penicillin V production was achieved in all engineered strains, with titres that were dependent on the version of the synthetic promoter used [356].

More recently, Mózsik et al. [357] applied the so-called “dead Cas9” (dCas9) protein for the control of gene expression in *P. chrysogenum*. dCas9 refers to defective versions of the Cas9 protein that cannot cut target DNA but still can bind to it, so they can be used for transcriptional regulation [358]. The authors fused a dCas9 protein to the highly active tripartite activator VP64-p65-Rta (VPR). This dCas9-VPR fusion was introduced in *P. chrysogenum* along with a suitable sgRNA. As a result, in two different experiments, they achieved the targeted transcriptional activation of a fluorescent reporter gene under the control of the *penDE* gene promoter from the penicillin cluster, and more importantly, the targeted activation of the transcription factor MacR. The expression of MacR in turn activated the silent macrophorin biosynthetic gene cluster, thus inducing the production of antimicrobial macrophorins.

In addition, Mózsik et al. [318] have developed a modular synthetic biology toolkit to engineer *P. chrysogenum* for the optimized production of enzymes or metabolites of interest. The authors present 96 genetic elements such as natural and synthetic promoters, terminators, fluorescent reporters, selection markers, and regulatory and DNA-binding domains of transcriptional regulators. In addition, there are components for the implementation of different CRISPR-based technologies. The purpose is to have available many different genetic parts that can be assembled into complex multipartite constructs to be integrated in the genome or expressed from an AMA1-based autonomously replicating vector. New synthetic transcription units, which may include synthetic transcriptional regulation devices, encoding natural or fusion proteins, can be more rapidly assembled in a standardized and modular manner to obtain novel fungal cell factories.

In summary, the CRISPR/Cas9 technique, in combination with other techniques, will allow engineering *P. chrysogenum* in ways that have not yet been fully explored, which include, but are not limited to, the replacement of natural gene promoters in BGCs by engineered strong and adjustable promoters to improve production yields of endogenous or exogenous compounds [323], the engineering of modular enzymes such as PKS and NRPS by module swapping to obtain modified secondary metabolites (discussed in [235] and [359]), and the combination of biosynthetic genes from different organisms to produce structurally diverse “unnatural products” [360].

## 13. Epilogue

**Figure 4:**
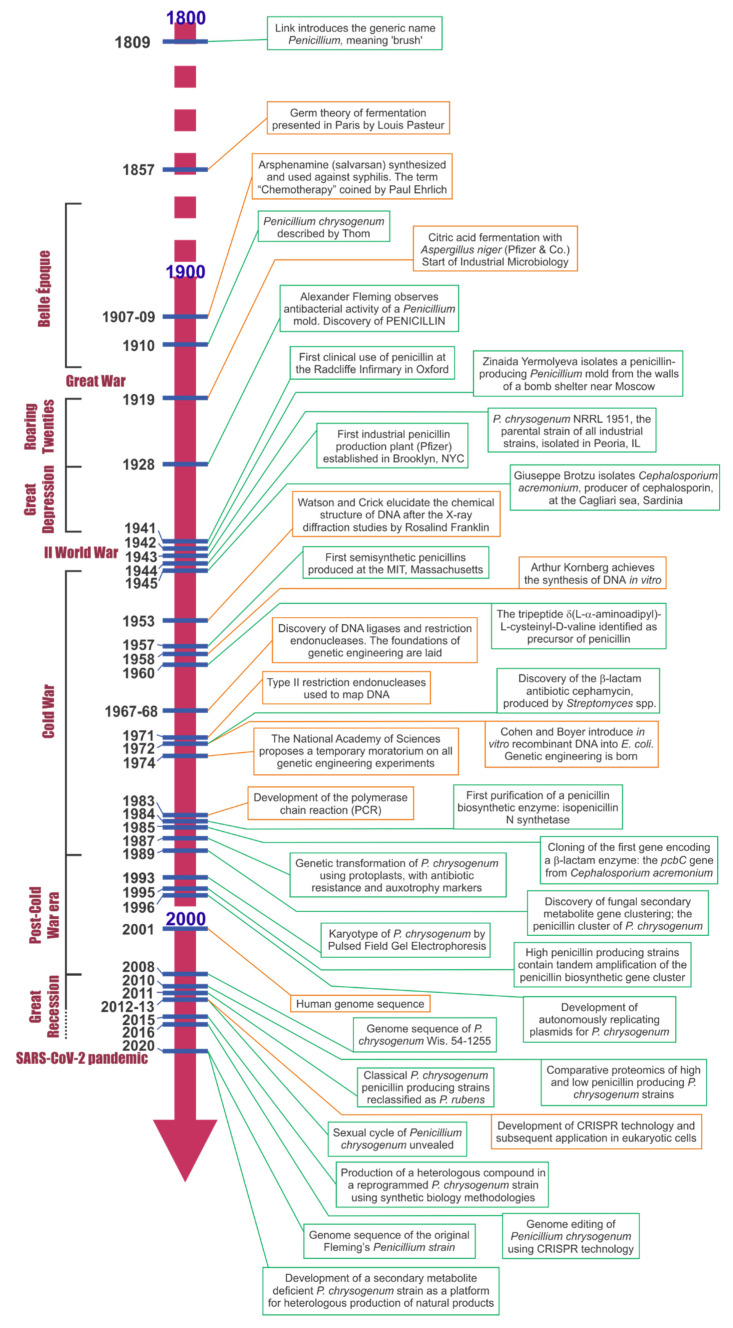
Brief history of the milestones in penicillin discovery, application, and research (green squares), intermingled with relevant scientific and technical developments that had an impact in the penicillin field, with a focus on genetic engineering (brown squares).

We have made a journey describing the main events surrounding penicillin discovery and the research developments in the study of its producer fungus, *P. chrysogenum*. Research has been mainly focused on improving the capacity of the fungus to produce high quantities of the antibiotic. Most of what has been described in this article concerns the lineage of *P. chrysogenum* strains derived from NRRL 1951, the ancestor of all industrial strains used for penicillin production, now reclassified as *P. rubens*. We have tried also to highlight new developments aiming to make the fungus into a platform for industrial production of heterologous metabolites by using synthetic biology approaches. Figure 4 summarizes these events.

We have also mentioned other *P. chrysogenum* strains regarding their capacity to produce a large variety of secondary metabolites and which were isolated from very diverse ecological niches (Table 1). Extending the field a little further, we can mention some additional biotechnological uses that are currently under investigation, such as the capacity of the Giza desert isolate *P. chrysogenum* MF318506 to produce nanoparticles with antimicrobial activities [361,362], the high bioremediation capacity against polychlorinated biphenyls (up to 96.5% removal) of two *P. chrysogenum* strains isolated from contaminated soils in France [363], and the use of an alginate-immobilized tyrosinase isolated from strain *P. chrysogenum* AUMC 14100 to remove phenol [364]. Additional biotechnology uses have been found for a mini-intein of only 157 amino acids present in the splicing-factor protein PRP8 of strain *P. chrysogenum* Pch-1 [365]. This mini-intein was recently used for expression of a bacterial phospholipase A2 in yeast with a strategy of intein-mediated delayed protein autoactivation to avoid the phospholipase toxic effect on the cell membrane [366]. *P. chrysogenum* strains have also been described as fungal endophytes promoting plant growth, as in the case of a *P. chrysogenum* root endophyte of Antarctic vascular plants, which was proposed by Oses-Pedraza et al. [367] to play a pivotal ecological role in Antarctic terrestrial ecosystems.

We could stop here, but it turns out *P. chrysogenum* shows its usefulness even after its death!. Dry mycelium of *P. chrysogenum* (DMP) constitutes the residual by-product of industry in penicillin production and does not contain penicillin or live mycelium. DMP has been shown to induce resistance against *Fusarium* wilt in melon and protect cucumber and tomato plants against the root-knot nematode *Meloidogyne javanica*, while an aqueous extract of DMP induces resistance in several crop species under both controlled and field conditions (see Li et al. [368] and references therein). DMP has been widely used in flue-cured tobacco planting in Yunnan province, China, yielding good economic and ecological benefits [369]. Recently, Li et al. [368] analyzed the molecular bases of the protective effect of the DMP polypeptide extract on tobacco plant against TMV infection, concluding that β-1,3-glucanase-mediated callose deposition and the ABA pathway seem to underlie the effect.

Penicillin was a “silver bullet”. In a world where infectious diseases were the primary cause of death, penicillin was a game changer. In addition to its own usefulness, it opened up the era of antibiotics, encouraging researchers to look avidly for more of those “magic bullets” produced by microorganisms. Today, we face new health threats: emerging diseases, with the potential to become pandemic, as we have recently experienced with the SARS-Cov-2, as well as old infectious diseases that re-emerge due to antibiotic resistance. The wealth of secondary metabolites still to be discovered and assayed should be an actor in the fight against them, and old *Penicillium chrysogenum* might also have something to contribute, perhaps a new chemotherapeutic agent, or through the heterologous production of new drugs against the new menaces.

## Figures and Tables

**Table 1:** Secondary metabolites isolated from different *P. chrysogenum* strains and their analyzed bioactivities.

Strain	Source	Secondary Metabolites	Bioactivity	Ref.
*P. notatum* isolate GWP A	Benchtop contamination	Rezishanones A-D (sorbicillinoids)	Weak activity against *Staphylococcus aureus* and *Bacillus subtilis*	[258]
*P. notatum* B-52	Salt sediments in Qinghai Lake, China	Di-hydrocitrinone (Isocoumarin deri-vative)	Inactive—evaluated against cell lines P388, BEL-7402, A-549 and HL-60	[259]
*P. notatum* B-52	Salt sediments in Qinghai Lake, China	Pennicitrinone A and D (Citrinin di-mers), citrinin and mycophenolic acid	Pennicitrinone A: weak cytotoxicity against cancer cell lines P388 and BEL-7402	[260]
*P. chrysogenum* QEN-24S	Unidentified marine red algal species of the genus *Laurencia*	Penicitides A and B (Polyketides). Glycerol derivatives 2-(2,4-dihydroxy-6-methylbenzoyl)-glycerol and penicimonoterpene	Penicitide A: cytotoxic activity against the human hepatocellular liver carcinoma (HepG2) cell line. Penicimonoterpene: potent activity against *Alternaria brassicae*	[261]
*P. chrysogenum* QEN-24S	Marine red algal species of the genus *Laurencia*	Penicisteroids A and B (polyoxygen-ated steroids)	Antifungal and cytotxic activity	[262]
*P. chrysogenum* QEN-24S	Marine red algal species of the genus *Laurencia*	Conidiogenones H and I (diterpenes)	Conidiation inducer	[263]
*P. chrysogenum* MFB574-2	Marine red alga Hypnea complex	Two polybrominated diphenyl ethers (1,1-diphenyl-2-picrylhydrazyl)	Free radical-scavenging activity	[264]
*P. chrysogenum* PXP-55	Surface of the roots of the mangrove plant *Rhizophora stylosa*, China	Chrysogesides A-E (cerebrosides) Chrysogedones A and B (2-pyridone alkaloids)	Chrysogeside B: antimicrobial activity against *Enterobacter aerogenes*. No compound showed cytotoxic effects on P388, HeLa, HL-60 or A549 cancer cell lines	[265]
*P. chrysogenum* IFL1	Agro-industrial residues, grape waste and cheese whey	Ciclopiazonic acid, rugulosin, formyl-xanthocilin X	Antimicrobial activities against bacteria, fungi and amoebae	[266]
*P. chrysogenum* PJX-17	Sediments collected in the South China Sea	Sorbicatechols A and B (polyketides)	Anti-influenza activity	[267]
*P. chrysogenum* HDN11-24	Rhizosphere soil of the mangrove plant *Acanthus ilicifolius*	Penicitols A-C (citrinin derivatives). Penixanacid A (xanthone, polyketide)	Cytotoxicity against HeLa, BEL-7402, HEK-293, HCT-116 and A549 cell lines	[268]
*P. chrysogenum* strains T04C and Fb	Walls of the tomb of King Tutankhamun in Upper Egypt	Pyomelanin	Contributes to survival of microorganisms in adverse conditions	[269]
*P. chrysogenum* isolate MS15	Leaf of olive tree *Olea europea*, Siwa oasis, Egyptian western desert	Meleagrin, roquefortine C, dehydro-histidyltryptophenyl-diketopiperazine (DHTD)	Meleagrin: c-Met inhibitory activity. Anti-tumoral activity against c-Met-dependent metastatic and invasive breast malignancies	[270]
*P. chrysogenum* V11	Endophytic, isolated from mangrove *Myoporum bontioides*	Penochalasins I and J (chaetoglobosins: cytochalasan alkaloids)	Penochalasin I: cytotoxicity against MDA-MB-435 and SGC-7901 human tumor cell lines. Penochalasin J: inhibited growth of *Colletotrichum gloeosporioides*	[271]
*P. chrysogenum* V11	Endophytic, isolated from mangrove *Myoporum bontioides* A. Gray	Penochalasins I and K. Chaetoglobosins A and C	Penochalasin K: inhibitory activities against *Colletotrichum gloeosporioides* and *Rhizoctonia solani*; cytotoxicity against cell lines MDA-MB-435 (breast cancer), SGC-7901 (gastric cancer) and A549 (lung adenocarcinoma)	[272]
*P. chrysogenum* SCSIO 41001	Deep sea sediment of Indian Ocean	Bipenicilisorin (isocoumarin dimer). Yaminterritrem C (merosesquiterpenoid). Penicitrinone F (citrinin dimer). Terremide D (alkaloid). δ valerolacton	Bipenicilisorin: cytotoxic activities against human cancer cell lines K562, A549, and Huh-7. Penicitrinone F: moderate inhibitory activity against cell line EV71	[273]
*P. chrysogenum* SCSIO 41001	Deep sea sediment of Indian Ocean	Four chrysines (chlorinated diphenyl ethers)	Inhibitory activity against α-glucosidase, delays absorption of glucose after a meal	[274]
*P. chrysogenum* MT-12	Endophytic, isolated from moss *Huperzia serrata*, Nanping, China	Chrysogenolides (A-H). Seven 3,5-dimethylorsellinic acid derived meroterpenoids	Several compounds inhibit NO production in LPS-activated RAW 264.7 macrophage cells	[275]
*P. chrysogenum* MT-12	Endophytic, isolated from moss *Huperzia serrata*, Nanping, China	12 Penicichrysogenins (polyketides)	Inhibition of nitric oxide production in lipopolysaccharide (LPS)-stimulated RAW264.7 macrophage cells	[276]
*P. chrysogenum* AD-1540	Inner tissue of the marine red alga *Grateloupia turuturu*	Chryxanthones A and B (benzophe-none derivatives)	Chryxanthone A: moderate cytotoxicity against BT-549 and HeLa cell lines. Chryxanthone B: selective growth-inhibitory effect on the A549 cell line	[277]
*P. chrysogenum* CHNSCLM-0019	Gorgonian *Dichotella gemmacea* collected in the South China Sea	Chrysopiperazines A and B, and Chrysopiperazine C (diketopiperazine alkaloids)	Inactive against several bacteria and *Candida albicans* at a concentration of 50 µM	[278]
*P. chrysogenum* MCCC 3A00292	Deep-sea sediment (2076 meters depth) of the South Atlantic Ocean	Peniciversiols A, B and C (versiol-type analogues). Penicilactones A and B (lactone derivatives)	Peniciversiol A: inhibitory effect against the BIU-87 cancer cell line	[279]
*P. chrysogenum* DXY-1	Marine sediments sur-rounding the East Sea, Taiwan Strait	Tyrosol (ethyl acetate extract)	Anti-quorum sensing (anti-QS) activity against *Chromobacterium violaceum* and *Pseudomonas aeruginosa*	[280]
*P. rubens* JGIPR9	Garden soil obtained from Madurai district, Tamil Nadu—India	Bioactive fraction P5 (containing indole-2, 3-(4,4-dimethyl-3-thiosemicarbazone)	Cytotoxic effect against HepG2, HeLa and MCF-7 cancer cells—induces apoptosis	[281]
*P. chrysogenum* SCSIO 07007	Deep-sea hydrothermal vent environments of the Western Atlantic	Chrysopyrones A and B (3,4,6-trisubstituted α-pyrone derivatives). Penilline C (indolyl diketopipe-razine derivative)	Chrysopyrones A and B: inhibitory activity against protein tyrosine phosphatase 1B (PTP1B), enzyme validated as biological target for Type II diabetes treatment	[282]
*P. chrysogenum* TJ403-CA4	Intestinal tract of the arthropod *Cryptotympana atrata*, collected in Lvliang City, Shanxi, China	Five 6−5−5−5-fused tetracyclic cyclopiane-type diterpenes (C20-carboxyl conidiogenone C, C20-carboxyl conidiogenone K, C19-hydroxy conidiogenone C, C7-hydroxy conidiogenone C, C8-hydroxy conidiogenone C)	Compounds 2 and 3 were active against methicillin-resistant *Staphylococcus aureus* (MRSA) ATCC 43300	[283]
*P. chrysogenum* 581F1	Marine sponge *Theonella swinhoei*, Xisha Islands, South China Sea	13-hydroxy-dihydrotrichodermolide and 10,11,27,28-tetrahydrotrisorbicillinone C (sorbicillinoids)	Biomolecular interactions targeting proteins GLP-1R (protein related to diabetes) and eEF2K (related inhibition tu-mor growth)	[284]

## Data Availability

Not applicable.
